# Supplementary material for: Associations between physical activity and asthma, eczema and obesity in children aged 12–16: an observational cohort study
Source: BMJ Open. 2019 Jan 20;9(1):e024858. doi: 10.1136/bmjopen-2018-024858 (PMC6340420; doi:10.1136/bmjopen-2018-024858)
Supplement: Supplementary file 1 [file bmjopen-2018-024858supp001.pdf]

SUPPLEMENTARY MATERIAL

Russell Jago, Ruth E. Salway, Andy R Ness, Julian P Hamilton-Shield, Matthew J Ridd, A John Henderson, *Associations between Physical Activity and Asthma, Eczema and Obesity in children aged 12-16*

List of Supplementary Tables

Table S1: Characteristics of imputed data

Table S2: Characteristics of observed data and missing values

Table S3: MVPA and sedentary time by health condition at each time point: unadjusted for confounders or other conditions

Table S4: Cross-sectional regression models for MVPA at average age 12, 14 and 16: minimally adjusted

List of Supplementary Figures

Figure S1: Venn diagrams of combinations of multiple conditions at ages 12, 14 and 16.

Figure S2: Observed average minutes of MVPA (left) and sedentary time (right) for different health conditions and by sex.

Table S1: Characteristics of imputed data

		Age 12 (n=5735)	Age 14 (n=4078)	Age 16 (n=2198)
		Mean (sd)	Mean (sd)	Mean (sd)
MVPA		57 (29)	52 (27)	47 (27)
Sedentary time		354 (73)	415 (81)	475 (84)
		%	%	%
**Condition**				
Asthma		24%	23%	27%
Eczema		21%	21%	27%
Overweight		14%	13%	13%
Obese		15%	13%	12%
**Combinations of conditions**				
None		47%	49%	44%
Asthma only		10%	11%	11%
Eczema only		9%	10%	12%
Obese only		8%	7%	6%
Asthma & Eczema		6%	6%	8%
Asthma & Obese		3%	3%	2%
Eczema & Obese		2%	2%	2%
Asthma, Eczema & Obese		2%	1%	2%
**Confounders**				
Sex (female)		53%	53%	55%
Mother's education:	None/CSE	11%	9%	8%
	Vocational	9%	8%	8%
	O level (exams at 16)	47%	47%	47%
	A level (exams at 18)	15%	16%	15%
	Degree	18%	20%	22%
Social class:	I Professional	13%	14%	16%
	II Manager & Tech	47%	49%	49%
	III Skilled, non-man	26%	25%	23%
	III Skilled, manual	9%	7%	7%
	IV Partly skilled	4%	4%	4%
	V Unskilled	1%	1%	1%

Table S2: Characteristics of observed data and missing values

		Age 12 (n=5735)		Age 14 (n=4078)		Age 16 (n=2198)	
		Mean (sd)	Missing (%)	Mean (sd)	Missing (%)	Mean (sd)	Missing (%)
MVPA		57 (29)	0 (0%)	52 (27)	0 (0%)	47 (27)	0 (0%)
Sedentary time		354 (73)	0 (0%)	415 (81)	0 (0%)	475 (84)	0 (0%)
		%		%		%	
**Condition**							
Asthma		24%	454 (8%)	23%	282 (7%)	27%	74 (3%)
Eczema		21%	842 (15%)	21%	542 (13%)	27%	114 (5%)
Overweight		14%	9 (0.2%)	13%	8 (0.2%)	13%	25 (1%)
Obese		15%	9 (0.2%)	13%	8 (0.2%)	12%	25 (1%)
**Combinations of conditions**							
None		55%	850 (15%)	56%	549 (13%)	51%	137 (6%)
Asthma only		13%		13%		13%	
Eczema only		11%		12%		14%	
Obese only		8%		7%		7%	
Asthma & Eczema		7%		7%		9%	
Asthma & Obese		3%		3%		2%	
Eczema & Obese		2%		2%		2%	
Asthma, Eczema & Obese		2%		1%		2%	
**Confounders**							
Sex (female)		53%	0 (0%)	53%	0 (0%)	55%	0 (0%)
Mother's education:	None/CSE	11%	230 (4%)	9%	136 (4%)	8%	71 (3%)
	Vocational	9%		8%		8%	
	O level (exams at 16)	47%		47%		47%	
	A level (exams at 18)	15%		16%		15%	
	Degree	18%		20%		22%	
Social class:	I Professional	13%	365 (6%)	14%	213 (5%)	16%	118 (5%)
	II Manager & Tech	47%		49%		49%	
	III Skilled, non-man	26%		25%		23%	
	III Skilled, manual	9%		7%		7%	
	IV Partly skilled	4%		4%		4%	
	V Unskilled	1%		1%		1%	

Table S3: MVPA and sedentary time by health condition at each time point: unadjusted for confounders or other conditions

	Age 12 (n=5735)					Age 14 (n=4078)					Age 16 (n=2198)				
	Mean	sd	Diff[1] (mins)	95% CI[1]	p-value[2]	Mean	sd	Diff[1] (mins)	95% CI[1]	p-value[2]	Mean	sd	Diff[1] (mins)	95% CI[1]	p-value[2]
**MVPA**															
Asthma	56	28	-0.47	(-2.29, 1.35)	0.613	53	27	1.25	(-0.79, 3.30)	0.229	46	29	-1.58	(-4.8, 1.02)	0.233
Eczema	54	26	-3.13	(-5.-8, -1.18)	0.002	51	27	-1.44	(-0.358, 0.70)	0.187	46	27	-0.62	(-3.20, 1.96)	0.638
Overweight	52	24	-5.63	(-7.81, -3.44)	<0.0005	50	27	-2.47	(-4.98, 0.03)	0.053	45	28	-1.64	(-5.04, 1.76)	0.344
Obese	51	25	-7.02	(-9.11, -4.94)	<0.0005	46	25	-6.02	(-8.51, -3.52)	<0.0005	42	25	-5.46	(-8.77, -2.15)	0.001
**Sedentary time**															
Asthma	349	72	-6.66	(-11.31, -2.02)	0.005	405	83	-13.15	(-19.31, -7.00)	<0.0005	474	89	-1.44	(-9.60, 6.72)	0.729
Eczema	355	72	1.12	(-3.86, 6.10)	0.660	418	82	4.11	(-2.60, 10.81)	0.229	477	82	2.82	(-5.22, 10.85)	0.492
Overweight	363	71	9.56	(4.04, 15.08)	0.001	424	86	10.61	(3.11, 18.11)	0.006	480	82	5.44	(-5.19, 16.08)	0.316
Obese	362	75	8.85	(3.57, 14.12)	0.001	428	79	15.26	(7.78, 22.73)	<0.0005	485	86	11.02	(0.67, 21.38)	0.037

[1] Diff and 95% CI are the mean difference between children with the condition and children without the condition and associated 95% confidence interval.

[2] P-value refers to t-test comparing those with the condition to those without.

Table S4: Cross-sectional regression models for MVPA at average age 12, 14 and 16: minimally adjusted

	Age 12 (n=5735)			Age 14 (n=4078)			Age 16 (n=2198)		
	Diff[3] (mins)	95 % CI	p-value	Diff[3] (mins)	95 % CI	p-value	Diff[3] (mins)	95 % CI	p-value
**MVPA**									
**All[1]**									
Asthma	-1.34	(-3.06, 0.38)	0.126	0.15	(-1.84, 2.14)	0.884	-2.59	(-5.12, -0.06)	0.045
Eczema	-1.95	(-3.78, -0.12)	0.037	-0.72	(-2.79, 1.35)	0.493	0.67	(-1.84, 3.17)	0.601
Overweight	-7.34	(-9.39, -5.28)	<0.0005	-3.10	(-5.50, -0.69)	0.012	-2.05	(-5.32, 1.22)	0.219
Obese	-8.64	(-10.61, -6.67)	<0.0005	-7.05	(-9.45, -4.65)	<0.0005	-5.15	(-8.35, -1.95)	0.002
**Boys[2]**									
Asthma	-1.95	(-4.68, 0.77)	0.161	0.66	(-2.50, 3.83)	0.680	-2.87	(-7.07, 1.33)	0.180
Eczema	-3.73	(-6.91, -0.54)	0.022	-0.21	(-3.76, 3.35)	0.910	2.03	(-2.42, 6.48)	0.371
Overweight	-11.16	(-14.60, -7.72)	<0.0005	-4.40	(-8.45, -0.35)	0.033	-4.64	(-10.33, 1.06)	0.110
Obese	-12.39	(-15.63, -9.14)	<0.0005	-10.00	(-13.87, -6.14)	<0.0005	-9.08	(-14.62, -3.54)	0.001
**Girls[2]**									
Asthma	-0.89	(-2.97, 1.20)	0.404	-0.56	(-3.01, 1.89)	0.656	-2.67	(-5.71, 0.37)	0.085
Eczema	-0.52	(-2.55, 1.51)	0.616	-1.19	(-3.76, 1.37)	0.361	-0.40	(-3.24, 2.44)	0.780
Overweight	-3.89	(-6.26, -1.53)	0.001	-1.90	(-4.74, 0.93)	0.188	0.07	(-3.70, 3.83)	0.972
Obese	-5.05	(-7.35, -2.76)	<0.0005	-4.11	(-7.06, -1.16)	0.006	-2.03	(-5.73, 1.67)	0.282
**Sedentary time**									
**All[1]**									
Asthma	-6.45	(-11.21, -1.69)	0.008	-12.07	(-18.25, -5.89)	<0.0005	-0.56	(-8.83, 7.72)	0.895
Eczema	1.69	(-3.38, 6.76)	0.513	4.62	(-2.05, 11.30)	0.174	1.81	(-6.30, 9.93)	0.661
Overweight	11.81	(6.26, 17.36)	<0.0005	12.70	(5.28, 20.12)	0.001	6.58	(-4.05, 17.21)	0.225
Obese	11.64	(6.32, 16.97)	<0.0005	18.96	(11.54, 26.37)	<0.0005	11.06	(0.66, 21.46)	0.037
**Boys[2]**									
Asthma	-6.84	(-13.42, -0.25)	0.042	-9.60	(-18.68, -0.52)	0.038	1.25	(-11.48, 13.98)	0.847
Eczema	3.96	(-3.43, 11.35)	0.294	4.63	(-5.47, 14.72)	0.368	-1.48	(-14.91, 11.95)	0.829
Overweight	13.19	(5.03, 21.36)	0.002	8.15	(-3.26, 19.56)	0.161	14.58	(-2.45, 31.61)	0.093
Obese	11.13	(3.443, 18.84)	0.005	26.34	(15.47, 37.22)	<0.0005	18.05	(1.48, 34.62)	0.033
**Girls[2]**									
Asthma	-5.92	(-12.65, 0.81)	0.085	-14.70	(-23.30, -6.10)	0.001	-1.51	(-12.39, 9.37)	0.786
Eczema	-0.25	(-7.14, 6.63)	0.943	4.50	(-4.41, 13.41)	0.321	4.36	(-5.97, 14.69)	0.408
Overweight	10.64	(3.06, 18.22)	0.006	16.48	(6.74, 26.22)	0.001	0.27	(-13.24, 13.77)	0.969
Obese	12.06	(4.68, 19.44)	0.001	11.83	(1.69, 21.98)	0.022	5.58	(-7.71, 18.86)	0.410

[1] Model adjusted for child age and sex. [2] Model adjusted for child age

[3] Coefficients indicate difference in minutes of MVPA/sedentary time between a child with and without the condition.

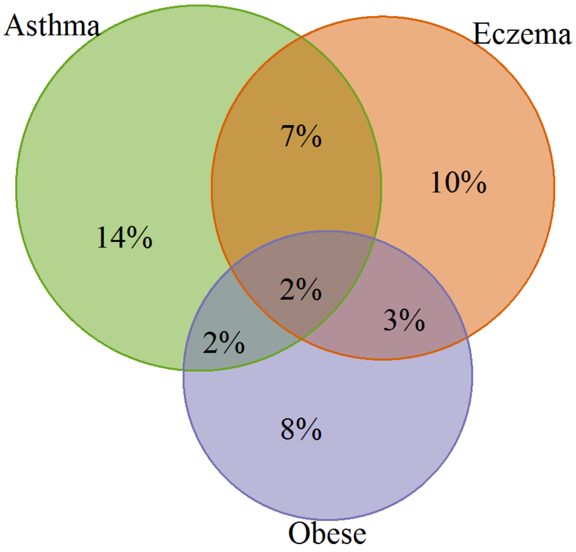

(a) Age 12

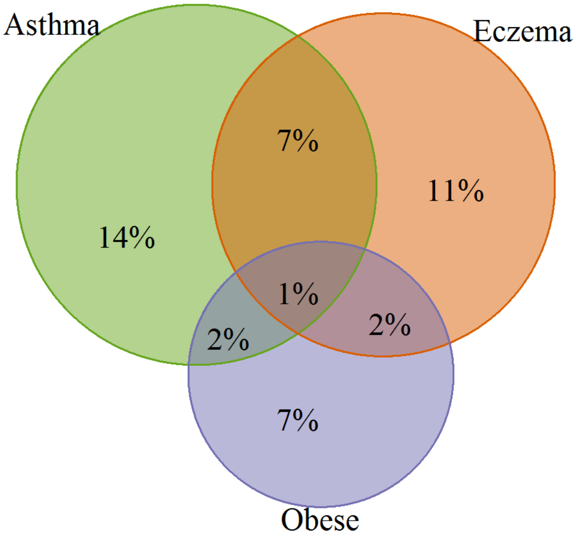

(b) Age 14

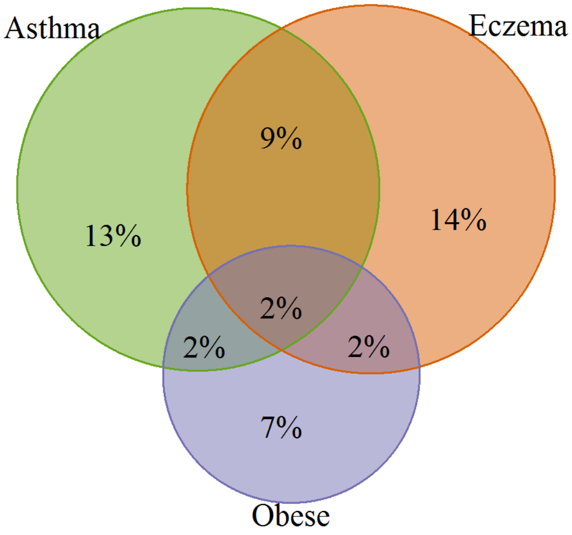

(c) Age 16

Figure S1: Venn diagrams of combinations of multiple conditions at ages 12, 14 and 16.

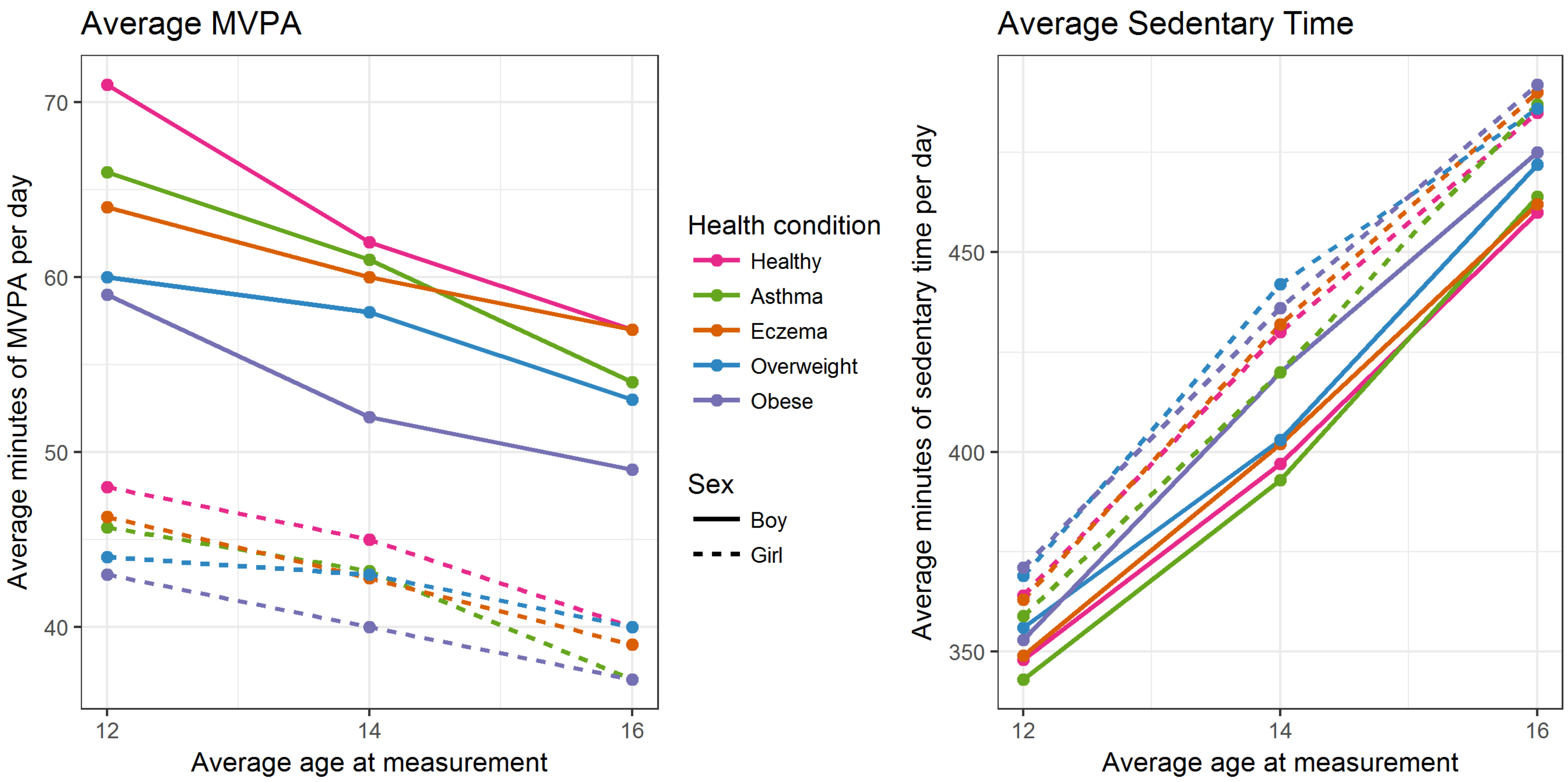

Figure S2: Observed average minutes of MVPA (left) and sedentary time (right) for different health conditions and by sex.
