# Supplementary material for: Associations between physical activity and asthma, eczema and obesity in children aged 12–16: an observational cohort study
Source: BMJ Open. 2019 Jan 20;9(1):e024858. doi: 10.1136/bmjopen-2018-024858 (PMC6340420; doi:10.1136/bmjopen-2018-024858)
Supplement: Author's manuscript [file bmjopen-2018-024858.draft_revisions.pdf]

BMJ Open is committed to open peer review. As part of this commitment we make the peer review history of every article we publish publicly available.

When an article is published we post the peer reviewers' comments and the authors' responses online. We also post the versions of the paper that were used during peer review. These are the versions that the peer review comments apply to.

The versions of the paper that follow are the versions that were submitted during the peer review process. They are not the versions of record or the final published versions. They should not be cited or distributed as the published version of this manuscript.

BMJ Open is an open access journal and the full, final, typeset and author-corrected version of record of the manuscript is available on our site with no access controls, subscription charges or pay-per-view fees (http://bmjopen.bmj.com).

If you have any questions on BMJ Open's open peer review process please email info.bmjopen@bmj.com

BMJ Open

ASSOCIATIONS BETWEEN PHYSICAL ACTIVITY AND ASTHMA, ECZEMA AND OBESITY IN CHILDREN AGED 12-16: AN OBSERVATIONAL COHORT STUDY

Journal:	*BMJ Open*
Manuscript ID	bmjopen-2018-024858
Article Type:	Research
Date Submitted by the Author:	18-Jun-2018

Complete List of Authors:	Jago, Russ; University of Bristol, Centre for Exercise, Nutrition and Health Sciences<br>Salway, Ruth; University of Bristol, Centre for Exercise, Nutrition and Health Sciences<br>Ness, Andy; School of Oral Dental Science,<br>Shield, Julian; University of Bristol, Biomedical Research Unit in Nutrition<br>Ridd, Matthew; University of Bristol, Social & Community Medicine; University of Bristol<br>Henderson, A. John; University of Bristol , Population Health Sciences

Keywords:	ALSPAC, Physical Activity, Obesity, Eczema < DERMATOLOGY, Asthma < THORACIC MEDICINE, cohort

SCHOLARONE™
Manuscripts

ASSOCIATIONS BETWEEN PHYSICAL ACTIVITY AND ASTHMA, ECZEMA AND OBESITY IN CHILDREN AGED 12-16: AN OBSERVATIONAL COHORT STUDY

Russell Jago PhD[1, 2, *], Ruth E. Salway PhD[1], Andy R Ness[3, 4], Julian P Hamilton-Shield[3], Matthew J Ridd [5], A John Henderson[6]

[1] Centre for Exercise, Nutrition & Health Sciences, School for Policy Studies, University of Bristol

[2] National Institute for Health Research (NIHR) Collaboration for Leadership in Applied Health Research and Care West (CLAHRC West) at University Hospitals Bristol NHS Foundation Trust

[3] NIHR Biomedical Research Centre at the University Hospitals Bristol NHS Foundation Trust and the University of Bristol

[4] Bristol Dental School, University of Bristol

[5] Centre for Academic Primary Care, Population Health Sciences, Bristol Medical School, University of Bristol

[6] Population Health Sciences, Bristol Medical School, University of Bristol

[*] **Address for correspondence:** Professor Russell Jago
Centre for Exercise, Nutrition & Health Sciences, School for Policy Studies
University of Bristol, 8 Priory RD, Bristol, BS8 1TZ
Email: russ.jago@bristol.ac.uk
Telephone: +44 (0)117 9546603
Fax: +44 (0) 117 954 6756

Word count: **Manuscript** (excluding tables) = 3470 words **Abstract**=295 words

For peer review only - http://bmjopen.bmj.com/site/about/guidelines.xhtml

ABSTRACT

Objectives: To compare the physical activity of adolescents with three common long-term conditions (asthma, eczema and obesity) to adolescents without these conditions.

Design: Cross-sectional and longitudinal analyses of adolescents at ages 12, 14 and 16 in a large UK cohort study

Setting: The Avon Longitudinal Study of Parents and Children.

Participants: 6473 adolescents with complete accelerometer data at at least one time point.

Methods: Mean minutes of moderate-to-vigorous-intensity physical activity (MVPA) and sedentary time per day were derived from accelerometer-based measurements at age 12, 14 and 16. Obesity was defined at each time point from height and weight measurements. Parents reported doctor-assessed asthma or eczema. Cross-sectional and longitudinal regression models examined any differences in MVPA or sedentary time for adolescents with asthma, eczema or obesity compared to those without.

Results: In longitudinal models, boys engaged in an average of 69.7 (95% CI: 67.6 to 71.7) mins MVPA at age 12, declining by 3.1 (95% CI: 2.6 to 3.6) mins/year while girls' average MVPA was 47.5 (95% CI: 46.1 to 48.9) mins at age 12, declining by 1.8 (95% CI: 1.5 to 2.1) mins/year. There was no strong evidence of differences in physical activity patterns of those with and without asthma or eczema. Obese boys engaged in 11.1 (95% CI: 8.7 to 13.6) fewer minutes of MVPA, and obese girls in 5.0 (95% CI: 3.3 to 6.8) fewer minutes than their non-obese counterparts. Cross-sectional models showed comparable findings.

Conclusions: Mean minutes of MVPA per day did not differ between adolescents with asthma or eczema and those without, but obese adolescents engaged in fewer minutes of MVPA. Findings reinforce the need for strategies to help obese adolescents be more active

For peer review only - http://bmjopen.bmj.com/site/about/guidelines.xhtml

but suggest no need to develop bespoke physical activity strategies for adolescents with mild asthma or eczema.

Keywords: ALSPAC, Physical Activity, Obesity, Eczema, asthma, cohort, children

For peer review only

For peer review only - http://bmjopen.bmj.com/site/about/guidelines.xhtml

ARTICLE SUMMARY

Strengths and limitations of this study

Strengths

- objective accelerometer-based assessment of physical activity at ages, 12, 14 and 16
- multiple assessments of the physical conditions asthma, eczema and obesity
- large representative population-based cohort allows longitudinal analysis of change over time

Limitations

- asthma and eczema assessments do not exactly coincide with the physical activity data and so we use lifetime prevalence rather than current asthma/eczema indicators.
- we are unable to differentiate between mild and severe asthma or eczema – more severe cases tend to be more persistent and it may be that persistence is linked to activity.

For peer review only - http://bmjopen.bmj.com/site/about/guidelines.xhtml

INTRODUCTION

Obesity, eczema (synonyms: atopic eczema/dermatitis) and asthma are three of the most prevalent childhood diseases in the UK. Data from the 2015/2016 National Child Measurement Programme showed that 9% of 4-5 year-olds and 20% of 10-11 year-olds in England were obese[1]. It has been estimated that 20% of children are diagnosed with eczema[2] and 9% with asthma[3], and children with eczema often have asthma. These long-term conditions place substantial burden on quality of life and healthcare expenditure. Understanding factors associated with the prevalence of these conditions is important to aid the development of prevention and management strategies.

Physical activity is a modifiable lifestyle factor that has been associated with adiposity[4] and asthma[5] in children, and adults with asthma engage in lower levels of physical activity than those without[6]. Low levels of physical activity reduce the quality of life in childhood and increase the risk of other co-morbidities[7]. Physical inactivity may be a problem among children with long-term conditions. There is some evidence that physical activity levels are lower among children with asthma than children without asthma. Children with eczema may be less active than children without eczema, due to reluctance to sweat which can irritate eczematous skin, but the current evidence base is very weak[8]. It may be that tailored strategies are needed to help those with asthma and eczema be more physically active, but the development of such approaches would only be warranted if there were evidence of lower physical activity.

There is some evidence to show that physical activity levels are lower among children who are overweight or obese[9]. While there have been many studies that have looked at the

For peer review only - http://bmjopen.bmj.com/site/about/guidelines.xhtml

association between physical activity and obesity these have tended to focus on cross-sectional surveys, or national surveys that have looked at associations over time but not within participants over time, for which cohort studies are essential. An analysis of the ALSPAC cohort reported a graded inverse association between physical activity and obesity at age 12[10]; an increase of 15 minutes of moderate to vigorous intensity physical activity (MVPA) per day was associated with 10% lower fat mass in girls and 12% lower in boys[11]. However, the authors used an ALSPAC-specific definition of MVPA[12] and reported an average MVPA of 20 minutes per day while the majority of current studies use the Evenson cut-points[13] which a comparison study reported was the reliable threshold[14] for young people. This difference is important as other studies that use Evenson cut-points typically report MVPA in the range of 50 to 70 minutes per day[15-17] and as such it is important to have data from ALSPAC that have been processed using the Evenson cut-points to facilitate international comparisons.

The overall objective of this paper is to examine the physical activity of adolescents with three of the most common long-term conditions and how they may change as they move through adolescence, using objective assessments of physical activity. This evidence is needed to identify whether there are important differences between children with different long-term conditions. The specific aims were: 1) to compare mean time spent sedentary and engaged in MVPA for adolescents with asthma, eczema and obesity at ages 12, 14 and 16; and 2) to examine prospective associations between sedentary and MVPA time from age 12 to 16 for those with and without these conditions.

METHODS

For peer review only - http://bmjopen.bmj.com/site/about/guidelines.xhtml

The Avon Longitudinal Study of Parents and Children (ALSPAC) is a birth cohort study based in the former county of Avon (UK)[18-20]. A total of 15247 pregnant women with expected delivery dates between April 1991 and December 1992 were recruited, with 14701 children alive at 1 year of age. Ethical approval for the study was obtained from the ALSPAC Ethics and Law Committee and the Local Research Ethics Committees, and written informed consent was obtained for all mothers, with parents providing written informed consent for young people's participation. Please note that the study website contains details of all the data that is available through a fully searchable data dictionary (http://www.bris.ac.uk/alspac/researchers/data-access/data-dictionary/).

Accelerometer Data

Adolescents wore an Actigraph AM7164 (Actigraph LLC, Fort Walton Beach, FL, USA) accelerometer for seven days. Accelerometer data were processed using Kinesoft (v3.3.75; Kinesoft, Saskatchewan, Canada) and analysis was restricted to those who provided at least three days of valid data. A valid day was defined as at least 500 minutes of data, after excluding intervals of ≥60 minutes of zero counts allowing up to two minutes of interruptions. The average number of MVPA (≥2296 counts per minute (CPM)) and sedentary minutes per day (<100 CPM) were derived for each adolescent based on the Evenson thresholds[13]. Accelerometer data with over 11715 CPM, fewer than 10 sedentary minutes or zero MVPA were considered erroneous and excluded from the analysis (16, 41 and 21 cases at ages 12, 14 and 16 respectively)[21].

Exposures

Height and weight were measured at the same time as the accelerometer data. Body mass index (BMI) was used to create age- and sex-specific overweight and obesity indicators using 85th and 95th percentiles based on UK BMI reference charts[22]. Throughout this paper the overweight category comprises adolescents who were overweight but not obese. The mother

For peer review only - http://bmjopen.bmj.com/site/about/guidelines.xhtml

was asked 'Has a doctor ever told you that your child has asthma?' at ages 8, 11 and 14, and 'Has a doctor ever told you that your child has eczema?' at ages 11 and 14. These were combined to form lifetime doctor-diagnosed asthma and eczema prevalence: 'has asthma ever been reported as diagnosed by a doctor?' and 'has eczema ever been reported as diagnosed by a doctor?', assessed by age 11 and 14. As these ages do not correspond to accelerometer collection, current asthma/eczema assessments were unavailable.

Confounders

The child's sex as recorded at birth and the age of the child at the accelerometer data collection were included as confounders. Mother's highest education was combined from data at 32w gestation and age 8. Household social class was calculated as the highest occupational social class reported by mother or partner from data at 18-weeks' gestation (both) and ages 4 (mother) and 8 (partner). Puberty was assessed from yearly questionnaires. For girls, an indicator of onset of menarche was used. For boys, Tanner stages based on pubic hair was used, as self-report data on Tanner stages for genitals has been found to be unreliable[23].

Statistical Analysis

Cross-sectional data were analysed at three timepoints corresponding to accelerometer data at average ages of 12, 14 and 16. We included any child with at least 3 valid days of accelerometer data. Obesity and overweight were calculated at the same timepoints. Asthma and eczema diagnoses did not correspond to the same timepoints, and so were taken from the closest prior measurement. Mother's highest education and household social class were calculated as described above and the same value used for all analyses. Cross-sectional analysis was based on 5735, 4078 and 2198 cases for ages 12, 14 and 16 respectively. For the

For peer review only - http://bmjopen.bmj.com/site/about/guidelines.xhtml

longitudinal analyses we combined data from all three timepoints, resulting in 6473 cases with data for at least one timepoint, and 1619 (25%) at all three.

Multiple imputation was used to account for missing data. For the cross-sectional analyses data were imputed for each dataset separately. For the longitudinal analysis, data were imputed if the child had accelerometer data for that timepoint, but not between timepoints, although data from other timepoints was used to impute missing data. Multiple imputation methods were used to create 20 imputed datasets at each timepoint for boys and girls separately, using 20 cycles of regression switching and combined regression coefficients across datasets using Rubin's rules[24]. All exposures, outcomes and confounders were included.

Cross-sectional Analyses

For each age, characteristics were described separately for all adolescents, those with asthma, eczema, who were overweight or obese, and for adolescents with none of the conditions. Average minutes of MVPA and sedentary time were compared between those with health conditions and those without using t-tests. Regression models were fitted for MVPA and sedentary minutes for all adolescents, and for boys and girls separately. Models include the asthma, eczema, overweight and obese indicators, and child age, sex, mother's education and household social class as confounders. For comparative purposes, minimally adjusted models which adjusted for just child age and sex were also run and are shown in supplementary tables.

Longitudinal Analysis

Random effect models were fitted to identify changes in physical activity measures over time between the health conditions, accounting for individual variability in measurements. As the

For peer review only - http://bmjopen.bmj.com/site/about/guidelines.xhtml

cross-sectional analyses suggest different patterns among boys and girls, all models were run separately by sex. We included indicators for asthma, eczema, obesity and overweight, and age at time of clinic to capture linear changes over time. All models were adjusted for mother's highest education and household social class. In this model, the constant term captures the average physical activity of a healthy child (i.e. with none of the health conditions) at age 12 for average confounder values. The age coefficient estimates a linear change in physical activity (or sedentary time) over time, which is the same for all health conditions, and additional terms for each health condition reflect any difference in the baseline activity.

A model was also fitted to assess if there were interactions between the health conditions to assess whether there was any additional effect associated with having multiple conditions. Finally, a model which allowed the change in physical activity over time to depend on health condition; so, for example, MVPA for an adolescent with asthma might decrease at a different rate between ages 12 and 16 than for an adolescent without was run. As self-reported pubertal status is susceptible to error[25] and pubertal status could be linked to obesity[26], pubertal status was considered in a secondary sensitivity analysis. All analyses were performed in Stata version 15.0[27].

RESULTS

Supplementary Table S1 summarises the characteristics of the imputed data, with summaries for observed data reported in Supplementary Table S2. Missing data ranged from <1% (obesity) to 15% (eczema). Between 24% and 27% of adolescents had ever been diagnosed with asthma, between 21% and 27% had ever been diagnosed with eczema, and between 13% and 15% were obese. Between 13% and 15% had more than one condition, with the most

For peer review only - http://bmjopen.bmj.com/site/about/guidelines.xhtml

common combination being asthma and eczema (between 7% and 9% of adolescents). The combinations at each age are summarised graphically in Supplementary Figure S1. The average minutes of MVPA per day decreased from 57 mins at age 12 to 47 mins at age 16, with average sedentary minutes increasing from 354 mins to 475 mins. Supplementary Figure S2 illustrates how MVPA and sedentary time changes with age among adolescents with the different conditions.

Cross-sectional Analyses

Supplementary Table S3 summarises the MVPA and sedentary time for adolescents with asthma, eczema, overweight and obese conditions, and for those with no health condition. Tables 1 and 2 present the regression models for MVPA and sedentary time respectively, adjusting for age, sex, mother's education and household social class, and report the difference between an adolescent with and without each health condition. These models include all conditions, including combinations; differences are additive if an adolescent has multiple conditions. A minimally adjusted model (Supplementary Table S4), adjusting only for child age and sex, shows very little difference to the coefficients reported in the full model.

The largest and most consistent differences are for adolescents who are overweight or obese. MVPA was 3.1-7.5 minutes lower among overweight adolescents, and 5.3- 8.9 minutes lower among obese adolescents. Similarly, sedentary time was 8.6-13.8 minutes higher among overweight adolescents, and 14.1-22.1 minutes higher among obese adolescents. These associations tend to be slightly stronger in boys than girls. Patterns for adolescents with asthma and eczema are weaker and less consistent. Associations between MVPA and asthma were inconclusive, with a lower MVPA (2.6 mins) among asthma sufferers at age 16, but no

For peer review only

For peer review only - http://bmjopen.bmj.com/site/about/guidelines.xhtml

other ages, and no differences when examining boys and girl separately. There was some association between asthma and sedentary time, with lower sedentary time at ages 12 and 14 (by 5.1 mins and 10.8 mins respectively), although evidence is inconclusive when

For peer review only

For peer review only - http://bmjopen.bmj.com/site/about/guidelines.xhtml

Table 1: Cross-sectional regression models for MVPA at average age 12, 14 and 16

	Age 12 (n=5735)			Age 14 (n=4078)			Age 16 (n=2198)		
	Diff[2]	95 % CI	p-value	Diff[2]	95 % CI	p-value	Diff[2]	95 % CI	p-value
**All[1]**									
Asthma	-1.47	(-3.19, 0.25)	0.094	-0.05	(-2.05, 1.95)	0.960	-2.62	(-5.16, -0.09)	0.043
Eczema	-1.85	(-3.68, -0.01)	0.049	-0.62	(-2.69, 1.44)	0.554	0.74	(-1.75, 3.24)	0.559
Overweight	-7.54	(-9.59, -5.48)	<0.0005	-3.12	(-5.53, -0.72)	0.011	-2.27	(-5.55, 1.01)	0.175
Obese	-8.94	(-10.92, -6.96)	<0.0005	-7.45	(-9.87, -5.04)	<0.0005	-5.29	(-8.50, -2.07)	0.001
**Boys[1]**									
Asthma	-2.03	(-4.76, 0.70)	0.144	0.47	(-2.71, 3.65)	0.773	-3.22	(-7.42, 0.98)	0.133
Eczema	-3.52	(-6.70, -0.34)	0.030	0.05	(-3.51, 3.61)	0.977	2.28	(-2.16, 6.71)	0.314
Overweight	-11.39	(-14.83, -7.95)	<0.0005	-4.47	(-8.54, -0.40)	0.031	-5.22	(-10.95, 0.50)	0.074
Obese	-12.52	(-15.77, -9.26)	<0.0005	-10.24	(-14.13, -6.35)	<0.0005	-9.42	(-14.99, -3.85)	0.001
**Girls[1]**									
Asthma	-1.01	(-3.09, 1.06)	0.339	-0.71	(-3.16, 1.75)	0.573	-2.48	(-5.52, 0.56)	0.110
Eczema	-0.49	(-2.52, 1.55)	0.639	-1.17	(-3.75, 1.41)	0.372	-0.34	(-3.16, 2.49)	0.816
Overweight	-4.07	(-6.44, -1.70)	0.001	-1.95	(-4.79, 0.88)	0.177	0.19	(-3.59, 3.96)	0.923
Obese	-5.39	(-7.70, -3.07)	<0.0005	-4.52	(-7.49, -1.55)	0.003	-1.88	(-5.59, 1.84)	0.321

[1] Models include all conditions, and are adjusted for child age, sex, mother's education and household social class as confounders.
[2] Difference in MVPA between a child with and without the condition, with all other variables the same.

For peer review only

For peer review only - http://bmjopen.bmj.com/site/about/guidelines.xhtml

Table 2: Cross-sectional regression models for sedentary time at average age 12, 14 and 16

	Age 12 (n=5735)			Age 14 (n=4078)			Age 16 (n=2198)		
	Diff[2]	95 % CI	p-value	Diff[2]	95 % CI	p-value	Diff[2]	95 % CI	p-value
**All[1]**									
Asthma	-5.14	(-9.80, -0.49)	0.030	-10.77	(-16.90, -4.65)	0.001	0.89	(-7.30, 9.09)	0.831
Eczema	0.76	(-4.25, 5.78)	0.765	4.02	(-2.55, 10.59)	0.230	0.95	(-7.09, 9.00)	0.816
Overweight	13.82	(8.334, 19.30)	<0.0005	13.53	(6.17, 20.88)	<0.0005	8.63	(-1.96, 19.22)	0.110
Obese	14.86	(9.59, 20.13)	<0.0005	22.06	(14.68, 29.43)	<0.0005	14.11	(3.75, 24.48)	0.008
**Boys[1]**									
Asthma	-5.86	(-12.33, 0.60)	0.075	-8.17	(-17.15, 0.81)	0.074	3.47	(-9.06, 16.01)	0.587
Eczema	2.64	(-4.63, 9.90)	0.476	2.80	(-7.19, 12.80)	0.582	-2.30	(-15.58, 10.98)	0.734
Overweight	15.07	(7.03, 23.12)	<0.0005	9.49	(-1.78, 20.75)	0.099	16.70	(-0.21, 33.62)	0.053
Obese	13.44	(5.82, 21.05)	0.001	29.28	(18.49, 40.06)	<0.0005	21.78	(5.32, 38.25)	0.010
**Girls[1]**									
Asthma	-4.41	(-11.00, 2.19)	0.190	-13.26	(-21.81, -4.72)	0.002	-0.69	(-11.54, 10.17)	0.901
Eczema	-0.85	(-7.67, 5.96)	0.806	4.55	(-4.29, 13.40)	0.312	3.33	(-6.97, 13.63)	0.526
Overweight	12.69	(5.19, 20.19)	0.001	17.43	(7.74, 27.12)	<0.0005	1.87	(-11.66, 15.41)	0.782
Obese	15.89	(8.55, 23.23)	<0.0005	14.44	(4.30, 24.58)	0.005	7.63	(-5.70, 20.96)	0.262

[1] Models include all conditions, and are adjusted for child age, sex, mother's education and household social class as confounders.

[2] Difference in sedentary time between a child with and without the condition.

For peer review only - http://bmjopen.bmj.com/site/about/guidelines.xhtml

considering boys and girls separately. There was very little association between eczema and either MVPA or sedentary time. We saw a slightly lower MVPA (3.5 mins) among boys at age 12, but no other ages, and no associations with sedentary time. It was not possible to explore associations with combinations of health conditions, due to small numbers (<100) for some combinations. However, there was no evidence of any additional association with having multiple conditions. So, for example, the difference between obese children with asthma and obese children without asthma is the same as the difference between non-obese children with asthma and non-obese children without.

Longitudinal Analysis

Table 3 summarizes the longitudinal models for MVPA and sedentary time, for boys and girls separately. The baseline represents a typical child at age 12 with the most common mother's highest education (O level – exams at age 16), and household class (Managerial & Technical). These coefficients are additive, so, for example, the average number of minutes of MVPA for a 12-year-old obese boy is estimated as 69.7-11.1 = 58.6 mins. Figure 1 plots the estimated average MVPA time and sedentary time for the different health conditions by age, for boys and girls.

The largest association was between physical activity and obesity, especially among boys. Asthma and eczema showed much weaker associations. A typical boy with no health conditions engaged in an average of 72.8 mins of MVPA at age 12, declining at a rate of 3.1 mins/year with age. They spent an average of 318.5 mins in sedentary time at age 12, increasing at a rate of 28.1 mins/year. Girls' average MVPA was lower at 49.3 mins at age 12, declining at a rate of 1.8 mins/year, while sedentary time was higher, with an average of 333.8 mins at age 12, increasing at a rate of 32.0 mins/year. We found no association between

For peer review only - http://bmjopen.bmj.com/site/about/guidelines.xhtml

Table 3: Comparison of longitudinal models for average minutes of MVPA and sedentary time per day

	Boys (2510 boys, 4847 measurements)			Girls (2744 girls, 5481 measurements)		
	Estimate	95% CI	P value	Estimate	95% CI	P value
**Average MVPA minutes per day**						
Baseline [1] (mins)	69.68	(67.64, 71.71)	<0.0005	47.52	(46.13, 48.90)	<0.0005
Change with age (mins/year)	-3.09	(-3.578, -2.61)	<0.0005	-1.78	(-2.10, -1.45)	<0.0005
Asthma[2] (mins)	-1.24	(-3.35, 0.87)	0.249	-1.19	(-2.81, 0.43)	0.150
Eczema[2] (mins)	-1.22	(-3.62. 1.17)	0.316	-0.87	(-2.52, 0.79)	0.305
Overweight[2] (mins)	-8.05	(-10.46, -5.63)	<0.0005	-2.33	(-3.99, -0.68)	0.006
Obese[2] (mins)	-11.12	(-13.57, -8.68)	<0.0005	-5.01	(-6.78, -3.25)	<0.0005
**Average sedentary minutes per day**						
Baseline [1] (mins)	346.64	(341.19, 352.08)	<0.0005	365.73	(31.05, 370.41)	<0.0005
Change with age (mins/year)	28.10	(26.94, 29.26)	<0.0005	31.97	(30.92, 33.03)	<0.0005
Asthma[2] (mins)	-4.06	(-9.84, 1.73)	0.169	-6.45	(-11.92, -0.98)	0.021
Eczema[2] (mins)	2.16	(-4.17, 8.49)	0.504	1.98	(-3.54, 7.50)	0.482
Overweight[2] (mins)	14.51	(8.38, 20.63)	<0.0005	12.63	(7.14, 18.12)	<0.0005
Obese[2] (mins)	17.37	(10.97, 23.78)	<0.0005	15.19	(9.28, 21.09)	<0.0005

[1] Baseline represents the average number of minutes for a healthy individual at age 11 with mother's highest education of O level (exam at age 16) and household social class of Managerial & Technical (II)
[2] Change from baseline if child has this health condition

For peer review only

For peer review only - http://bmjopen.bmj.com/site/about/guidelines.xhtml

asthma and either MVPA or sedentary time for boys. Among girls, there was no association between MVPA and asthma, but asthma sufferers spent 6.5 fewer minutes in sedentary time than non-sufferers. We found no association between eczema and either MVPA or sedentary time for boys or girls.

Overweight and obese adolescents had lower MVPA and higher sedentary time. Associations are stronger for boys than for girls, and stronger for obesity than overweight. Being overweight is associated with a decrease in MVPA of 8.1 mins and an increase in sedentary time of 14.5 mins for boys, compared to a decrease of 2.3 mins in MVPA and an increase of 12.6 mins in sedentary time for girls. Obesity is associated with a decrease in MVPA of 11.1 mins and an increase in sedentary time of 17.4 mins among boys, and a decrease in MVPA of 5.0 mins and an increase in sedentary time of 15.2 mins among girls.

There was no evidence of any additional association with combinations of multiple conditions. This suggests that any associations between physical activity and health conditions may be additive, although low prevalence of multiple conditions in this sample mean that we lacked statistical power to explore this fully. There was no evidence that the rate of change in physical activity over time differed between health condition. Including puberty in the longitudinal model (results not shown) reduced the strength of association with obesity and being overweight slightly but had no effect on associations with asthma or eczema.

DISCUSSION

In this paper we found no strong evidence of differences in the mean minutes of MPVA or sedentary time per day between adolescents with asthma or eczema and those without these

For peer review only - http://bmjopen.bmj.com/site/about/guidelines.xhtml

conditions in the cross-sectional models. We found a small association between asthma and fewer minutes of sedentary time among girls, but no associations for MVPA or eczema. Results suggest that adolescents who are overweight or obese engage in lower levels of physical activity at ages 12, 14 and 16, with stronger associations for boys than girls. These findings were confirmed in the longitudinal models which showed lower levels of MVPA and higher sedentary time with increasing levels of obesity. Specifically, obese boys engage in 11.1 fewer minutes of MVPA and 17.4 more sedentary minutes compared to a boy of similar age and demographics, while obese girls engage in 5.0 fewer minutes of MVPA and 15.2 more sedentary minutes than their non-obese counterparts. This finding is consistent with a previous Mendelian randomization analysis which suggested a likely bi-directional association between physical activity and adiposity among young people[28].

The lack of an association between physical activity, sedentary time and asthma challenges a body of research which has reported that children with asthma are less active than their peers. However, previous literature has relied on relatively small cross-sectional studies using self-reported assessments of physical activity and asthma[29-32]. This analysis uses a population-based rather than clinical cohort and therefore contains a lower proportion of children with moderate to severe asthma. Also, because the study combines data measured at different times, we report lifetime asthma prevalence rather than current asthma. Therefore, caution should be exercised in any direct comparison with other studies. Low physical activity and fitness have been suggested as risk factors for asthma onset[33] and exercise has been shown to improve markers of asthma control[34], which appear inconsistent with our findings. Concerns that exercise-induced bronchoconstriction might limit physical activity in children with controlled asthma appear to be ill-founded[35]. Our results therefore provide reassurance that

For peer review only - http://bmjopen.bmj.com/site/about/guidelines.xhtml

adolescents with asthma in a general population take part in similar levels of physical activity to their peers and there is no need for condition-specific behaviour change programs.

A previous Mendelian randomisation study from the ALSPAC cohort at age 7 identified obesity as a risk factor for asthma[36]. This is consistent with our results. We observed a higher prevalence of obesity amongst asthma sufferers (20%, 16% and 17% at ages 12, 14 and 16) than those without asthma (14%, 12% and 12% respectively). Furthermore, obesity with asthma has been implicated as a risk for more frequent asthma exacerbations[37], disease severity and a reduced response to therapy[38]. Some small studies have suggested that weight loss may aid asthma management in children and adults[39-41]. These studies and the consistent reporting of associations between asthma, obesity prevalence and disease morbidity supported by this study suggest that larger and longer weight management trials in obese individuals with asthma are warranted.

Previous evidence of any difference in the physical activity patterns of children with and without eczema was unclear[8]. There is some evidence of an association in adults[42], and possibly an association with severe eczema in children[43]. There may also be a link between eczema and obesity in North America and Asia, although there is no evidence of an association in Europe[44]. Our analyses found no differences in accelerometer-measured physical activity for those with lifetime-diagnosed eczema of any severity, which is encouraging given the psychosocial impact of even mild eczema. While parents may restrict activities such as swimming because of concerns about the effects on the child's skin[45], our findings suggest that the presence of eczema does not overall negatively impact physical activity in this group and therefore there is no need for eczema-specific physical activity programs.

For peer review only - http://bmjopen.bmj.com/site/about/guidelines.xhtml

Strengths and limitations

The main strength of this study is the objective assessment of physical activity at ages, 12, 14 and 16 and multiple assessments of physical conditions on a large cohort of adolescents. Although only 25% participants had data at all three timepoints, 61% had data for at least two, making the longitudinal analysis reasonably robust, although the study was not powered to test for interactions between conditions. We used multiple imputation to minimize the amount of missing data but recognize that there are limitations of this approach, including the underlying assumption that data are missing at random[46 47].

The study combines data measured at different times. Specifically, the asthma and eczema assessments do not exactly coincide with the physical activity data and so we were unable to include current asthma/eczema indicators. We also have no asthma/eczema information between 11 and 14 and so cannot determine, for example, if asthma precedes obesity. Finally, we are unable to differentiate between mild and severe asthma or eczema – more severe cases tend to be more persistent and it may be that persistence is linked to activity. The data were collected approximately a decade ago and it is possible that physical activity patterns and the prevalence of physical conditions, especially obesity, may have changed over this period.

CONCLUSIONS

Analysis of the ALSPAC population-based cohort has shown that physical activity and sedentary time did not differ for adolescents with asthma or eczema and those without, but obese adolescents engaged in fewer minutes of MVPA and more sedentary time. Findings

For peer review only - http://bmjopen.bmj.com/site/about/guidelines.xhtml

reinforce the need for strategies to help obese adolescents be more active but suggest no need to develop bespoke physical activity strategies for adolescents with mild asthma or eczema.

ACKNOWLEDGEMENTS

We are extremely grateful to all the families who took part in this study, the midwives for their help in recruiting them, and the whole ALSPAC team, which includes interviewers, computer and laboratory technicians, clerical workers, research scientists, volunteers, managers, receptionists and nurses.

FUNDING

The UK Medical Research Council and Wellcome (Grant ref: 102215/2/13/2) and the University of Bristol provide core support for ALSPAC. This publication is the work of the authors and AJH, ARN, RES and RJ will serve as guarantors for the contents of this paper. A comprehensive list of grants funding is available on the ALSPAC website. Physical activity data collection was supported by the US National Heart, Lung, and Blood Institute (R01 HL071248-01A). RJ is partly supported by the National Institute for Health Research (NIHR) Collaboration for Leadership in Applied Health Research and Care West (CLAHRC West) at University Hospitals Bristol NHS Foundation Trust. The NIHR Biomedical Research Centre at University Hospitals Bristol NHS Foundation Trust and the University of Bristol supports ARN and JPH-S. MR is funded by a National Institute for Health Research Post-Doctoral Research Fellowship (PDF-2014-07-013).

COMPETING INTERESTS: none

For peer review only

CONTRIBUTORS

Conception/design: all

Methodology: RJ, RES, MJR, AJH

Data analysis: RES

Drafting: RJ, RES

Revising: all

Final approval: all

DATA SHARING STATEMENT

This is a secondary data analysis based on data from the ALSPAC cohort. The access policy for the ALSPAC data can be found at http://www.bristol.ac.uk/media-library/sites/alspac/documents/researchers/data-access/ALSPAC_Access_Policy.pdf

DISCLAIMER

The views expressed in this publication are those of the author(s) and not necessarily those of the NHS, the National Institute for Health Research or the Department of Health.

For peer review only

For peer review only - http://bmjopen.bmj.com/site/about/guidelines.xhtml

REFERENCES

1. National Statistics. National Child Measurement Programme, England 2015-2016 school year. 2016
2. Flohr C, Mann J. New insights into the epidemiology of childhood atopic dermatitis. *Allergy* 2014;69(1):3-16. doi: 10.1111/all.12270
3. Asthma UK. Asthma facts and statistics 2017 [Available from: https://www.asthma.org.uk/about/media/facts-and-statistics/ accessed 13/03/2017.
4. Reichert FF, Baptista Menezes AM, Wells JC, et al. Physical activity as a predictor of adolescent body fatness: a systematic review. *Sports Med* 2009;39(4):279-94. doi: 10.2165/00007256-200939040-00002
5. Lochte L, Nielsen KG, Petersen PE, et al. Childhood asthma and physical activity: a systematic review with meta-analysis and Graphic Appraisal Tool for Epidemiology assessment. *BMC Pediatr* 2016;16:50. doi: 10.1186/s12887-016-0571-4
6. Cordova-Rivera L, Gibson PG, Gardiner PA, et al. A systematic review of associations of physical activity and sedentary time with asthma outcomes. *J Allergy Clin Immunol Pract* 2018 doi: 10.1016/j.jaip.2018.02.027 [published Online First: 2018/03/07]
7. World Health Organization. Global health risks: mortality and burden of disease attributable to selected major risks: World Health Organization, , 2009.
8. Kim A, Silverberg JI. A systematic review of vigorous physical activity in eczema. *Br J Dermatol* 2016;174(3):660-2. doi: 10.1111/bjd.14179
9. Coombs NA, Stamatakis E. Associations between objectively assessed and questionnaire-based sedentary behaviour with BMI-defined obesity among general population children and adolescents living in England. *BMJ open* 2015;5(6):e007172. doi: 10.1136/bmjopen-2014-007172
10. Ness AR, Leary SD, Mattocks C, et al. Objectively Measured Physical Activity and Fat Mass in a Large Cohort of Children. *PLoS Med* 2007;4(3):e97.
11. Riddoch CJ, Leary SD, Ness AR, et al. Prospective associations between objective measures of physical activity and fat mass in 12-14 year old children: the Avon Longitudinal Study of Parents and Children (ALSPAC). *BMJ* 2009;339:b4544. doi: 10.1136/bmj.b4544 [published Online First: 2009/11/28]
12. Mattocks C, Leary S, Ness A, et al. Calibration of an accelerometer during free-living activities in children. *Int J Pediatr Obes* 2007:1-9.
13. Evenson KR, Catellier DJ, Gill K, et al. Calibration of two objective measures of physical activity for children. *J Sports Sci* 2008;26(14):1557-65. doi: 1080/02640410802334196.
14. Trost SG, Loprinzi PD, Moore R, et al. Comparison of Accelerometer Cut-points for Predicting Activity Intensity in Youth. *Med Sci Sports Exerc* 2011;43(7)
15. Cooper AR, Goodman A, Page AS, et al. Objectively measured physical activity and sedentary time in youth: the International children's accelerometry database (ICAD). *Int J Behav Nutr Phys Act* 2015;12:113. doi: 10.1186/s12966-015-0274-5
16. Harding SK, Page AS, Falconer C, et al. Longitudinal changes in sedentary time and physical activity during adolescence. *Int J Behav Nutr Phys Act* 2015;12:44. doi: 10.1186/s12966-015-0204-6
17. Jago R, Solomon-Moore E, Macdonald-Wallis C, et al. Change in children's physical activity and sedentary time between Year 1 and Year 4 of primary school in the B-PROACT1V cohort. *Int J Behav Nutr Phys Act* 2017;14(1):33. doi: 10.1186/s12966-017-0492-0

For peer review only - http://bmjopen.bmj.com/site/about/guidelines.xhtml

18. Golding J, Pembrey M, Jones R. ALSPAC--the Avon Longitudinal Study of Parents and Children. I. Study methodology. *Paediatr Perinat Epidemiol* 2001;15(1):74-87.
19. Boyd A, Golding J, Macleod J, et al. Cohort Profile: the 'children of the 90s'--the index offspring of the Avon Longitudinal Study of Parents and Children. *Int J Epidemiol* 2013;42(1):111-27. doi: 10.1093/ije/dys064 [published Online First: 2012/04/18]
20. Fraser A, Macdonald-Wallis C, Tilling K, et al. Cohort Profile: The Avon Longitudinal Study of Parents and Children: ALSPAC mothers cohort. *International Journal of Epidemiology* 2013;42:97-110.
21. Adams MA, Johnson WD, Tudor-Locke C. Steps/day translation of the moderate-to-vigorous physical activity guideline for children and adolescents. *Int J Behav Nutr Phys Act* 2013;10:49. doi: 10.1186/1479-5868-10-49 [published Online First: 2013/04/23]
22. Cole TJ, Freeman JV, Preece MA. Body mass index reference curves for the UK, 1990. *Arch Dis Child* 1995;73(1):25-9.
23. Monteilh C, Kieszak S, Flanders WD, et al. Timing of maturation and predictors of Tanner stage transitions in boys enrolled in a contemporary British cohort. *Paediatr Perinat Epidemiol* 2011;25(1):75-87. doi: 10.1111/j.1365-3016.2010.01168.x [published Online First: 2010/12/08]
24. Rubin DB. Multiple imputation after 18+ years. *Journal of the American Statistical Association* 1996;91(434):473-789.
25. Monteilha C, Kieszaka S, Flanders WD, et al. Timing of maturation and predictors of Tanner stage transitions in boys enrolled in a contemporary British cohort. *Paediatric and Perinatal Epidemiology* 2011;25:75-87. doi: 10.1111/j.1365-3016.2010.01168.x
26. Kaplowitz PB. Link Between Body Fat and the Timing of Puberty. *Pediatrics* 2008;121(Supplement 3)
27. Stata Statistical Software: Release 15 [program]. College Station, TX: StataCorp LLC, 2017.
28. Richmond RC, Davey Smith G, Ness AR, et al. Assessing causality in the association between child adiposity and physical activity levels: a Mendelian randomization analysis. *PLoS Med* 2014;11(3):e1001618. doi: 10.1371/journal.pmed.1001618 [published Online First: 2014/03/20]
29. Cassim R, Koplin JJ, Dharmage SC, et al. The difference in amount of physical activity performed by children with and without asthma: A systematic review and meta-analysis. *J Asthma* 2016;53(9):882-92. doi: 10.1080/02770903.2016.1175474
30. Glazebrook C, McPherson AC, Macdonald IA, et al. Asthma as a barrier to children's physical activity: implications for body mass index and mental health. *Pediatrics* 2006;118(6):2443-9. doi: 10.1542/peds.2006-1846
31. Lam KM, Yang YH, Wang LC, et al. Physical Activity in School-Aged Children with Asthma in an Urban City of Taiwan. *Pediatr Neonatol* 2016;57(4):333-7. doi: 10.1016/j.pedneo.2015.05.003
32. Leinaar E, Alamian A, Wang L. A systematic review of the relationship between asthma, overweight, and the effects of physical activity in youth. *Ann Epidemiol* 2016;26(7):504-10 e6. doi: 10.1016/j.annepidem.2016.06.002 [published Online First: 2016/07/28]
33. Rasmussen F, Lambrechtsen J, Siersted HC, et al. Low physical fitness in childhood is associated with the development of asthma in young adulthood: the Odense schoolchild study. *The European respiratory journal : official journal of the European Society for Clinical Respiratory Physiology* 2000;16(5):866-70. [published Online First: 2001/01/12]

For peer review only - http://bmjopen.bmj.com/site/about/guidelines.xhtml

34. Lucas SR, Platts-Mills TA. Physical activity and exercise in asthma: relevance to etiology and treatment. *J Allergy Clin Immunol* 2005;115(5):928-34. doi: 10.1016/j.jaci.2005.01.033 [published Online First: 2005/05/04]
35. Orenstein DM. Pulmonary problems and management concerns in youth sports. *Pediatr Clin North Am* 2002;49(4):709-21, v-vi. [published Online First: 2002/09/26]
36. Granell R, Henderson AJ, Evans DM, et al. Effects of BMI, fat mass, and lean mass on asthma in childhood: a Mendelian randomization study. *PLoS Med* 2014;11(7):e1001669. doi: 10.1371/journal.pmed.1001669
37. Ahmadizar F, Vijverberg SJ, Arets HG, et al. Childhood obesity in relation to poor asthma control and exacerbation: a meta-analysis. *The European respiratory journal : official journal of the European Society for Clinical Respiratory Physiology* 2016;48(4):1063-73. doi: 10.1183/13993003.00766-2016 [published Online First: 2016/09/03]
38. Forno E, Celedon JC. The effect of obesity, weight gain, and weight loss on asthma inception and control. *Curr Opin Allergy Clin Immunol* 2017;17(2):123-30. doi: 10.1097/ACI.0000000000000339 [published Online First: 2016/12/29]
39. Willeboordse M, van de Kant KDG, Tan FE, et al. A Multifactorial Weight Reduction Programme for Children with Overweight and Asthma: A Randomized Controlled Trial. *PLoS One* 2016;11(6):e0157158. doi: 10.1371/journal.pone.0157158 [published Online First: 2016/06/15]
40. Luna-Pech JA, Torres-Mendoza BM, Luna-Pech JA, et al. Normocaloric diet improves asthma-related quality of life in obese pubertal adolescents. *Int Arch Allergy Immunol* 2014;163(4):252-8. doi: 10.1159/000360398 [published Online First: 2014/04/10]
41. Adeniyi FB, Young T. Weight loss interventions for chronic asthma. *Cochrane Database Syst Rev* 2012(7):CD009339. doi: 10.1002/14651858.CD009339.pub2 [published Online First: 2012/07/13]
42. Silverberg JI, Song J, Pinto D, et al. Atopic Dermatitis Is Associated with Less Physical Activity in US Adults. *J Invest Dermatol* 2016;136(8):1714-6.
43. Strom MA, Silverberg JI. Associations of Physical Activity and Sedentary Behavior with Atopic Disease in United States Children. *J Pediatr* 2016;174:247-53.e3.
44. Zhang A, Silverberg JI. Association of atopic dermatitis with being overweight and obese: a systematic review and metaanalysis. *J Am Acad Dermatol* 2015;72(4):606-16. doi: 10.1016/j.jaad.2014.12.013
45. Santer M, Burgess H, Yardley Y, et al. Experiences of carers managing childhood eczema and their views on its treatment: a qualitative study. *Br J Gen Pract* 2012;62(597):e261-e26.
46. Sterne JA, White IR, Carlin JB, et al. Multiple imputation for missing data in epidemiological and clinical research: potential and pitfalls. *BMJ* 2009;338:b2393. doi: 10.1136/bmj.b2393
47. White IR, Royston P, Wood AM. Multiple imputation using chained equations: Issues and guidance for practice. *Stat Med* 2011;30(4):377-99. doi: 10.1002/sim.4067

For peer review only

For peer review only - http://bmjopen.bmj.com/site/about/guidelines.xhtml

FIGURE LEGENDS AND KEYS

Figure 1: Venn diagrams of combinations of multiple conditions at ages 12, 14 and 16.

Figure 2: Observed average minutes of MVPA (left) and sedentary time (right) for different health conditions and by sex.

Figure 3: Fitted models for average minutes of MVPA (left) and sedentary time (right) for different health conditions and by sex.

For peer review only

For peer review only - http://bmjopen.bmj.com/site/about/guidelines.xhtml

For peer review only

For peer review only - http://bmjopen.bmj.com/site/about/guidelines.xhtml

Table 7: Comparison of longitudinal models for average minutes of MVPA and sedentary time per day

	Boys (2510 boys, 4847 measurements)			Girls (2744 girls, 5481 measurements)		
	**Estimate**	**95% CI**	**P value**	**Estimate**	**95% CI**	**P value**
**Average MVPA minutes per day**						
Baseline [1] (mins)	69.68	(67.64, 71.71)	<0.0005	47.52	(46.13, 48.90)	<0.0005
Change with age (mins/year)	-3.09	(-3.578, -2.61)	<0.0005	-1.78	(-2.10, -1.45)	<0.0005
Asthma[2] (mins)	-1.24	(-3.35, 0.87)	0.249	-1.19	(-2.81, 0.43)	0.150
Eczema[2] (mins)	-1.22	(-3.62. 1.17)	0.316	-0.87	(-2.52, 0.79)	0.305
Overweight[2] (mins)	-8.05	(-10.46, -5.63)	<0.0005	-2.33	(-3.99, -0.68)	0.006
Obese[2] (mins)	-11.12	(-13.57, -8.68)	<0.0005	-5.01	(-6.78, -3.25)	<0.0005
**Average sedentary minutes per day**						
Baseline [1] (mins)	346.64	(341.19, 352.08)	<0.0005	365.73	(31.05, 370.41)	<0.0005
Change with age (mins/year)	28.10	(26.94, 29.26)	<0.0005	31.97	(30.92, 33.03)	<0.0005
Asthma[2] (mins)	-4.06	(-9.84, 1.73)	0.169	-6.45	(-11.92, -0.98)	0.021
Eczema[2] (mins)	2.16	(-4.17, 8.49)	0.504	1.98	(-3.54, 7.50)	0.482
Overweight[2] (mins)	14.51	(8.38, 20.63)	<0.0005	12.63	(7.14, 18.12)	<0.0005
Obese[2] (mins)	17.37	(10.97, 23.78)	<0.0005	15.19	(9.28, 21.09)	<0.0005

[1] Baseline represents the average number of minutes for a healthy individual at age 11 with mother's highest education of O level (exam at age 16) and household social class of Managerial & Technical (II)

[2] Change from baseline if child has this health condition

For peer review only

For peer review only

For peer review only - http://bmjopen.bmj.com/site/about/guidelines.xhtml

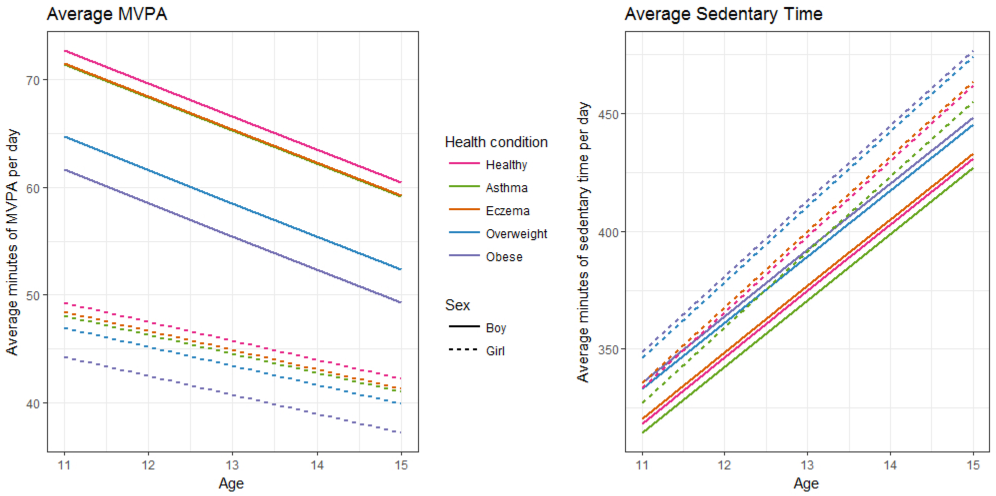

Figure 1: Estimated average MVPA and sedentary time for different health conditions by age for boys and girls

264x132mm (96 x 96 DPI)

SUPPLEMENTARY MATERIAL

Russell Jago, Ruth E. Salway, Andy R Ness, Julian P Hamilton-Shield, Matthew J Ridd, A John Henderson, *Associations between Physical Activity and Asthma, Eczema and Obesity in children aged 12-16*

List of Supplementary Tables

Table S1: Characteristics of imputed data

Table S2: Characteristics of observed data and missing values

Table S3: MVPA and sedentary time by health condition at each time point: unadjusted for confounders or other conditions

Table S4: Cross-sectional regression models for MVPA at average age 12, 14 and 16: minimally adjusted

List of Supplementary Figures

Figure S1: Venn diagrams of combinations of multiple conditions at ages 12, 14 and 16.

Figure S2: Observed average minutes of MVPA (left) and sedentary time (right) for different health conditions and by sex.

For peer review only

Table S1: Characteristics of imputed data

		Age 12 (n=5735)	Age 14 (n=4078)	Age 16 (n=2198)
		Mean (sd)	Mean (sd)	Mean (sd)
MVPA		57 (29)	52 (27)	47 (27)
Sedentary time		354 (73)	415 (81)	475 (84)
		%	%	%
**Condition**				
Asthma		24%	23%	27%
Eczema		21%	21%	27%
Overweight		14%	13%	13%
Obese		15%	13%	12%
**Combinations of conditions**				
None		47%	49%	44%
Asthma only		10%	11%	11%
Eczema only		9%	10%	12%
Obese only		8%	7%	6%
Asthma & Eczema		6%	6%	8%
Asthma & Obese		3%	3%	2%
Eczema & Obese		2%	2%	2%
Asthma, Eczema & Obese		2%	1%	2%
**Confounders**				
Sex (female)		53%	53%	55%
Mother's education:	None/CSE	11%	9%	8%
	Vocational	9%	8%	8%
	O level (exams at 16)	47%	47%	47%
	A level (exams at 18)	15%	16%	15%
	Degree	18%	20%	22%
Social class:	I Professional	13%	14%	16%
	II Manager & Tech	47%	49%	49%
	III Skilled, non-man	26%	25%	23%
	III Skilled, manual	9%	7%	7%
	IV Partly skilled	4%	4%	4%
	V Unskilled	1%	1%	1%

For peer review only

Table S2: Characteristics of observed data and missing values

		Age 12 (n=5735)		Age 14 (n=4078)		Age 16 (n=2198)	
		Mean (sd)	Missing (%)	Mean (sd)	Missing (%)	Mean (sd)	Missing (%)
MVPA		57 (29)	0 (0%)	52 (27)	0 (0%)	47 (27)	0 (0%)
Sedentary time		354 (73)	0 (0%)	415 (81)	0 (0%)	475 (84)	0 (0%)
		%		%		%	
**Condition**							
Asthma		24%	454 (8%)	23%	282 (7%)	27%	74 (3%)
Eczema		21%	842 (15%)	21%	542 (13%)	27%	114 (5%)
Overweight		14%	9 (0.2%)	13%	8 (0.2%)	13%	25 (1%)
Obese		15%	9 (0.2%)	13%	8 (0.2%)	12%	25 (1%)
**Combinations of conditions**							
None		55%	850 (15%)	56%	549 (13%)	51%	137 (6%)
Asthma only		13%		13%		13%	
Eczema only		11%		12%		14%	
Obese only		8%		7%		7%	
Asthma & Eczema		7%		7%		9%	
Asthma & Obese		3%		3%		2%	
Eczema & Obese		2%		2%		2%	
Asthma, Eczema & Obese		2%		1%		2%	
**Confounders**							
Sex (female)		53%	0 (0%)	53%	0 (0%)	55%	0 (0%)
Mother's education:	None/CSE	11%	230 (4%)	9%	136 (4%)	8%	71 (3%)
	Vocational	9%		8%		8%	
	O level (exams at 16)	47%		47%		47%	
	A level (exams at 18)	15%		16%		15%	
	Degree	18%		20%		22%	
Social class:	I Professional	13%	365 (6%)	14%	213 (5%)	16%	118 (5%)
	II Manager & Tech	47%		49%		49%	
	III Skilled, non-man	26%		25%		23%	
	III Skilled, manual	9%		7%		7%	
	IV Partly skilled	4%		4%		4%	
	V Unskilled	1%		1%		1%	

For peer review only

Table S3: MVPA and sedentary time by health condition at each time point: unadjusted for confounders or other conditions

	Age 12 (n=5735)					Age 14 (n=4078)					Age 16 (n=2198)				
	Mean	sd	Diff[1]	95% CI[1]	p-value[2]	Mean	sd	Diff[1]	95% CI[1]	p-value[2]	Mean	sd	Diff[1]	95% CI[1]	p-value[2]
**MVPA**															
Asthma	56	28	-0.47	(-2.29, 1.35)	0.613	53	27	1.25	(-0.79, 3.30)	0.229	46	29	-1.58	(-4.8, 1.02)	0.233
Eczema	54	26	-3.13	(-5.-8, -1.18)	0.002	51	27	-1.44	(-0.358, 0.70)	0.187	46	27	-0.62	(-3.20, 1.96)	0.638
Overweight	52	24	-5.63	(-7.81, -3.44)	<0.0005	50	27	-2.47	(-4.98, 0.03)	0.053	45	28	-1.64	(-5.04, 1.76)	0.344
Obese	51	25	-7.02	(-9.11, -4.94)	<0.0005	46	25	-6.02	(-8.51, -3.52)	<0.0005	42	25	-5.46	(-8.77, -2.15)	0.001
**Sedentary time**															
Asthma	349	72	-6.66	(-11.31, -2.02)	0.005	405	83	-13.15	(-19.31, -7.00)	<0.0005	474	89	-1.44	(-9.60, 6.72)	0.729
Eczema	355	72	1.12	(-3.86, 6.10)	0.660	418	82	4.11	(-2.60, 10.81)	0.229	477	82	2.82	(-5.22, 10.85)	0.492
Overweight	363	71	9.56	(4.04, 15.08)	0.001	424	86	10.61	(3.11, 18.11)	0.006	480	82	5.44	(-5.19, 16.08)	0.316
Obese	362	75	8.85	(3.57, 14.12)	0.001	428	79	15.26	(7.78, 22.73)	<0.0005	485	86	11.02	(0.67, 21.38)	0.037

[1] Diff and 95% CI are the mean difference between children with the condition and children without the condition and associated 95% confidence interval.

[2] P-value refers to t-test comparing those with the condition to those without.

For peer review only

Table S4: Cross-sectional regression models for MVPA at average age 12, 14 and 16: minimally adjusted

	Age 12 (n=5735)			Age 14 (n=4078)			Age 16 (n=2198)		
	Diff[3]	95 % CI	p-value	Diff[3]	95 % CI	p-value	Diff[3]	95 % CI	p-value
**MVPA**									
**All[1]**									
Asthma	-1.34	(-3.06, 0.38)	0.126	0.15	(-1.84, 2.14)	0.884	-2.59	(-5.12, -0.06)	0.045
Eczema	-1.95	(-3.78, -0.12)	0.037	-0.72	(-2.79, 1.35)	0.493	0.67	(-1.84, 3.17)	0.601
Overweight	-7.34	(-9.39, -5.28)	<0.0005	-3.10	(-5.50, -0.69)	0.012	-2.05	(-5.32, 1.22)	0.219
Obese	-8.64	(-10.61, -6.67)	<0.0005	-7.05	(-9.45, -4.65)	<0.0005	-5.15	(-8.35, -1.95)	0.002
**Boys[2]**									
Asthma	-1.95	(-4.68, 0.77)	0.161	0.66	(-2.50, 3.83)	0.680	-2.87	(-7.07, 1.33)	0.180
Eczema	-3.73	(-6.91, -0.54)	0.022	-0.21	(-3.76, 3.35)	0.910	2.03	(-2.42, 6.48)	0.371
Overweight	-11.16	(-14.60, -7.72)	<0.0005	-4.40	(-8.45, -0.35)	0.033	-4.64	(-10.33, 1.06)	0.110
Obese	-12.39	(-15.63, -9.14)	<0.0005	-10.00	(-13.87, -6.14)	<0.0005	-9.08	(-14.62, -3.54)	0.001
**Girls[2]**									
Asthma	-0.89	(-2.97, 1.20)	0.404	-0.56	(-3.01, 1.89)	0.656	-2.67	(-5.71, 0.37)	0.085
Eczema	-0.52	(-2.55, 1.51)	0.616	-1.19	(-3.76, 1.37)	0.361	-0.40	(-3.24, 2.44)	0.780
Overweight	-3.89	(-6.26, -1.53)	0.001	-1.90	(-4.74, 0.93)	0.188	0.07	(-3.70, 3.83)	0.972
Obese	-5.05	(-7.35, -2.76)	<0.0005	-4.11	(-7.06, -1.16)	0.006	-2.03	(-5.73, 1.67)	0.282
**Sedentary time**									
**All[1]**									
Asthma	-6.45	(-11.21, -1.69)	0.008	-12.07	(-18.25, -5.89)	<0.0005	-0.56	(-8.83, 7.72)	0.895
Eczema	1.69	(-3.38, 6.76)	0.513	4.62	(-2.05, 11.30)	0.174	1.81	(-6.30, 9.93)	0.661
Overweight	11.81	(6.26, 17.36)	<0.0005	12.70	(5.28, 20.12)	0.001	6.58	(-4.05, 17.21)	0.225
Obese	11.64	(6.32, 16.97)	<0.0005	18.96	(11.54, 26.37)	<0.0005	11.06	(0.66, 21.46)	0.037
**Boys[2]**									
Asthma	-6.84	(-13.42, -0.25)	0.042	-9.60	(-18.68, -0.52)	0.038	1.25	(-11.48, 13.98)	0.847
Eczema	3.96	(-3.43, 11.35)	0.294	4.63	(-5.47, 14.72)	0.368	-1.48	(-14.91, 11.95)	0.829
Overweight	13.19	(5.03, 21.36)	0.002	8.15	(-3.26, 19.56)	0.161	14.58	(-2.45, 31.61)	0.093
Obese	11.13	(3.443, 18.84)	0.005	26.34	(15.47, 37.22)	<0.0005	18.05	(1.48, 34.62)	0.033
**Girls[2]**									
Asthma	-5.92	(-12.65, 0.81)	0.085	-14.70	(-23.30, -6.10)	0.001	-1.51	(-12.39, 9.37)	0.786
Eczema	-0.25	(-7.14, 6.63)	0.943	4.50	(-4.41, 13.41)	0.321	4.36	(-5.97, 14.69)	0.408
Overweight	10.64	(3.06, 18.22)	0.006	16.48	(6.74, 26.22)	0.001	0.27	(-13.24, 13.77)	0.969
Obese	12.06	(4.68, 19.44)	0.001	11.83	(1.69, 21.98)	0.022	5.58	(-7.71, 18.86)	0.410

[1] Model adjusted for child age and sex.
[2] Model adjusted for child sex
[3] Coefficients indicate difference in MVPA/sedentary time between a child with and without the condition.

For peer review only

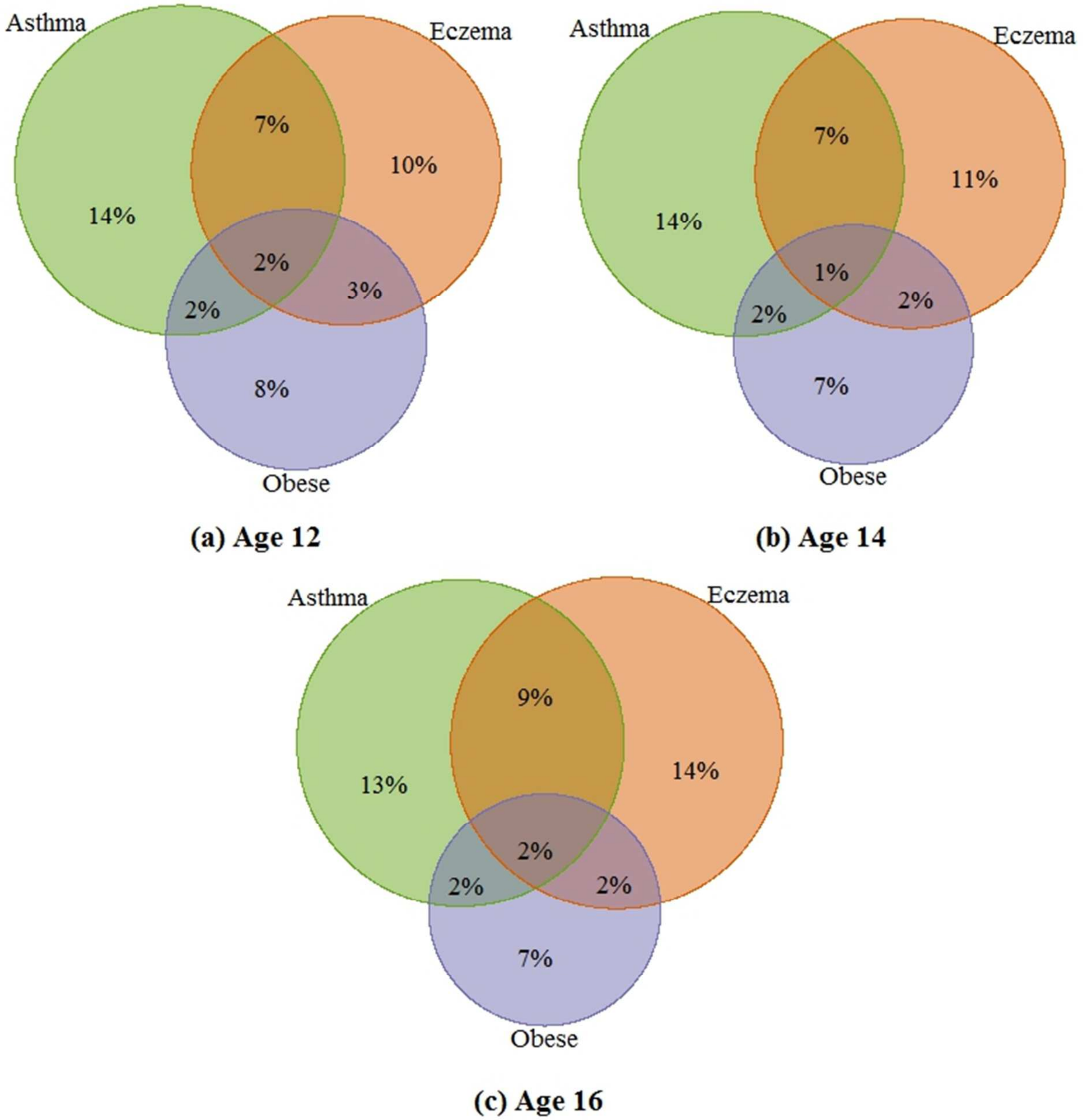

Figure S1: Venn diagrams of combinations of multiple conditions at ages 12, 14 and 16.

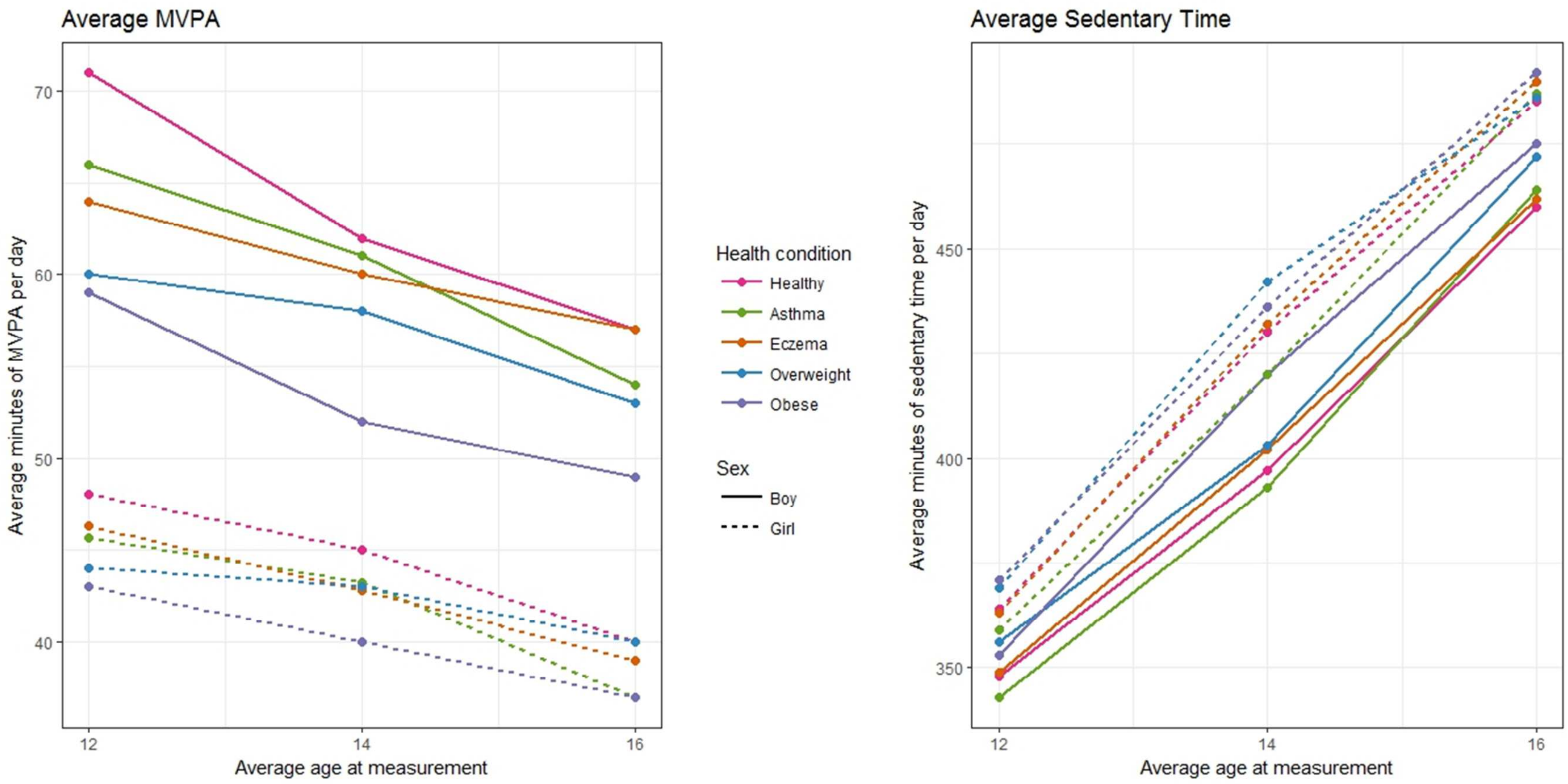

Figure S2: Observed average minutes of MVPA (left) and sedentary time (right) for different health conditions and by sex.

STROBE 2007 (v4) Statement—Checklist of items that should be included in reports of *cohort studies*

Section/Topic	Item #	Recommendation	Reported on page #
**Title and abstract**	1	(*a*) Indicate the study's design with a commonly used term in the title or the abstract	Title page
		(*b*) Provide in the abstract an informative and balanced summary of what was done and what was found	2-3
**Introduction**			
Background/rationale	2	Explain the scientific background and rationale for the investigation being reported	5-6
Objectives	3	State specific objectives, including any prespecified hypotheses	6
**Methods**			
Study design	4	Present key elements of study design early in the paper	6-7
Setting	5	Describe the setting, locations, and relevant dates, including periods of recruitment, exposure, follow-up, and data collection	6-7
Participants	6	(*a*) Give the eligibility criteria, and the sources and methods of selection of participants. Describe methods of follow-up	6-7
		(*b*) For matched studies, give matching criteria and number of exposed and unexposed	N/A
Variables	7	Clearly define all outcomes, exposures, predictors, potential confounders, and effect modifiers. Give diagnostic criteria, if applicable	7-8
Data sources/ measurement	8*	For each variable of interest, give sources of data and details of methods of assessment (measurement). Describe comparability of assessment methods if there is more than one group	7-8
Bias	9	Describe any efforts to address potential sources of bias	8-9
Study size	10	Explain how the study size was arrived at	8
Quantitative variables	11	Explain how quantitative variables were handled in the analyses. If applicable, describe which groupings were chosen and why	9-10
Statistical methods	12	(*a*) Describe all statistical methods, including those used to control for confounding	9-10
		(*b*) Describe any methods used to examine subgroups and interactions	9-10
		(*c*) Explain how missing data were addressed	9
		(*d*) If applicable, explain how loss to follow-up was addressed	n/a
		(*e*) Describe any sensitivity analyses	10
**Results**			

For peer review only

Participants	13*	(a) Report numbers of individuals at each stage of study—eg numbers potentially eligible, examined for eligibility, confirmed eligible, included in the study, completing follow-up, and analysed	8, 10
		(b) Give reasons for non-participation at each stage	n/a
		(c) Consider use of a flow diagram	
Descriptive data	14*	(a) Give characteristics of study participants (eg demographic, clinical, social) and information on exposures and potential confounders	10-11
		(b) Indicate number of participants with missing data for each variable of interest	10
		(c) Summarise follow-up time (eg, average and total amount)	n/a
Outcome data	15*	Report numbers of outcome events or summary measures over time	10
Main results	16	(*a*) Give unadjusted estimates and, if applicable, confounder-adjusted estimates and their precision (eg, 95% confidence interval). Make clear which confounders were adjusted for and why they were included	11-15
		(*b*) Report category boundaries when continuous variables were categorized	n/a
		(*c*) If relevant, consider translating estimates of relative risk into absolute risk for a meaningful time period	n/a
Other analyses	17	Report other analyses done—eg analyses of subgroups and interactions, and sensitivity analyses	16
**Discussion**			
Key results	18	Summarise key results with reference to study objectives	16-18
**Limitations**			
Interpretation	20	Give a cautious overall interpretation of results considering objectives, limitations, multiplicity of analyses, results from similar studies, and other relevant evidence	17-19
Generalisability	21	Discuss the generalisability (external validity) of the study results	17-19
**Other information**			
Funding	22	Give the source of funding and the role of the funders for the present study and, if applicable, for the original study on which the present article is based	20

*Give information separately for cases and controls in case-control studies and, if applicable, for exposed and unexposed groups in cohort and cross-sectional studies.

Note: An Explanation and Elaboration article discusses each checklist item and gives methodological background and published examples of transparent reporting. The STROBE checklist is best used in conjunction with this article (freely available on the Web sites of PLoS Medicine at http://www.plosmedicine.org/, Annals of Internal Medicine at http://www.annals.org/, and Epidemiology at http://www.epidem.com/). Information on the STROBE Initiative is available at www.strobe-statement.org.

For peer review only

BMJ Open

ASSOCIATIONS BETWEEN PHYSICAL ACTIVITY AND ASTHMA, ECZEMA AND OBESITY IN CHILDREN AGED 12-16: AN OBSERVATIONAL COHORT STUDY

Journal:	*BMJ Open*
Manuscript ID	bmjopen-2018-024858.R1
Article Type:	Research
Date Submitted by the Author:	19-Oct-2018
Complete List of Authors:	Jago, Russ; University of Bristol, Centre for Exercise, Nutrition and Health Sciences<br>Salway, Ruth; University of Bristol, Centre for Exercise, Nutrition and Health Sciences<br>Ness, Andy; School of Oral Dental Science,<br>Shield, Julian; University of Bristol, Biomedical Research Unit in Nutrition<br>Ridd, Matthew; University of Bristol, Social & Community Medicine; University of Bristol<br>Henderson, A. John; University of Bristol , Population Health Sciences
**Primary Subject Heading**:	Paediatrics
Secondary Subject Heading:	Epidemiology
Keywords:	ALSPAC, Physical Activity, Obesity, Eczema < DERMATOLOGY, Asthma < THORACIC MEDICINE, cohort

SCHOLARONE™
Manuscripts

ASSOCIATIONS BETWEEN PHYSICAL ACTIVITY AND ASTHMA, ECZEMA AND OBESITY IN CHILDREN AGED 12-16: AN OBSERVATIONAL COHORT STUDY

Russell Jago PhD[1, 2, *], Ruth E. Salway PhD[1], Andy R Ness[3, 4], Julian P Hamilton-Shield[3], Matthew J Ridd [5], A John Henderson[6]

[1] Centre for Exercise, Nutrition & Health Sciences, School for Policy Studies, University of Bristol

[2] National Institute for Health Research (NIHR) Collaboration for Leadership in Applied Health Research and Care West (CLAHRC West) at University Hospitals Bristol NHS Foundation Trust

[3] NIHR Biomedical Research Centre at the University Hospitals Bristol NHS Foundation Trust and the University of Bristol

[4] Bristol Dental School, University of Bristol

[5] Centre for Academic Primary Care, Population Health Sciences, Bristol Medical School, University of Bristol

[6] Population Health Sciences, Bristol Medical School, University of Bristol

[*]**Address for correspondence:** Professor Russell Jago
Centre for Exercise, Nutrition & Health Sciences, School for Policy Studies
University of Bristol, 8 Priory RD, Bristol, BS8 1TZ
Email: russ.jago@bristol.ac.uk
Telephone: +44 (0)117 9546603
Fax: +44 (0) 117 954 6756

For peer review only

Word count: **Manuscript** (excluding tables) = 3470 words **Abstract**=295 words

For peer review only - http://bmjopen.bmj.com/site/about/guidelines.xhtml

ABSTRACT

Objectives: To compare the physical activity of adolescents with three common long-term conditions (asthma, eczema and obesity) to adolescents without these conditions.

Design: Cross-sectional and longitudinal analyses of adolescents at ages 12, 14 and 16 in a large UK cohort study.

Setting: The Avon Longitudinal Study of Parents and Children.

Participants: 6473 adolescents with complete accelerometer data at at least one time point.

Methods: Mean minutes of moderate-to-vigorous-intensity physical activity (MVPA) and sedentary time per day were derived from accelerometer-based measurements at age 12, 14 and 16. Obesity was defined at each time point from height and weight measurements. Parents reported doctor-assessed asthma or eczema. Cross-sectional and longitudinal regression models examined any differences in MVPA or sedentary time for adolescents with asthma, eczema or obesity compared to those without.

Results: In longitudinal models, boys engaged in an average of 69.7 (95% CI: 67.6 to 71.7) mins MVPA at age 12, declining by 3.1 (95% CI: 2.6 to 3.6) mins/year while girls' average MVPA was 47.5 (95% CI: 46.1 to 48.9) mins at age 12, declining by 1.8 (95% CI: 1.5 to 2.1) mins/year. There was no strong evidence of differences in physical activity patterns of those with and without asthma or eczema. Obese boys engaged in 11.1 (95% CI: 8.7 to 13.6) fewer minutes of MVPA, and obese girls in 5.0 (95% CI: 3.3 to 6.8) fewer minutes than their non-obese counterparts. Cross-sectional models showed comparable findings.

Conclusions: Mean minutes of MVPA per day did not differ between adolescents with asthma or eczema and those without, but obese adolescents engaged in fewer minutes of MVPA. Findings reinforce the need for strategies to help obese adolescents be more active

For peer review only - http://bmjopen.bmj.com/site/about/guidelines.xhtml

but suggest no need to develop bespoke physical activity strategies for adolescents with mild asthma or eczema.

Keywords: ALSPAC, Physical Activity, Obesity, Eczema, asthma, cohort, children

For peer review only

For peer review only - http://bmjopen.bmj.com/site/about/guidelines.xhtml

ARTICLE SUMMARY

Strengths and limitations of this study

Strengths

- Objective accelerometer-based assessment of physical activity at ages, 12, 14 and 16
- Multiple assessments of the physical conditions asthma, eczema and obesity
- Large representative population-based cohort allows longitudinal analysis of change over time

Limitations

- Asthma and eczema assessments do not exactly coincide with the physical activity data and so we use lifetime prevalence rather than current asthma/eczema indicators.
- We are unable to differentiate between mild and severe asthma or eczema – more severe cases tend to be more persistent and it may be that persistence is linked to activity.

For peer review only

INTRODUCTION

Obesity, eczema (synonyms: atopic eczema/dermatitis) and asthma are three of the most prevalent childhood long-term conditions in the UK. Data from the 2015/2016 National Child Measurement Programme showed that 9% of 4-5 year-olds and 20% of 10-11 year-olds in England were obese[1]. It has been estimated that 20% of children are diagnosed with eczema[2] and 9% with asthma[3], and children with eczema often have asthma. These long-term conditions place substantial burden on quality of life and healthcare expenditure. Understanding factors associated with the prevalence of these conditions is important to aid the development of prevention and management strategies.

Physical activity is a modifiable lifestyle factor that has been associated with adiposity[4] and asthma[5] in children, and adults with asthma engage in lower levels of physical activity than those without[6]. Low levels of physical activity reduce the quality of life in childhood and increase the risk of other co-morbidities[7]. Physical inactivity may be a problem among children with long-term conditions. There is some evidence that physical activity levels are lower among children with asthma than children without asthma [8]. Children with eczema may be less active than children without eczema, due to reluctance to sweat which can irritate eczematous skin, but the current evidence base is very weak[9]. It may be that tailored strategies are needed to help those with asthma and eczema be more physically active, but the development of such approaches would only be warranted if there were evidence of lower physical activity.

There is some evidence to show that physical activity levels are lower among children who are overweight or obese[10]. While there have been many studies that have looked at the association between physical activity and obesity these have tended to focus on cross-

For peer review only - http://bmjopen.bmj.com/site/about/guidelines.xhtml

sectional surveys, or national surveys that have looked at associations over time but not within participants over time, for which cohort studies are essential. An analysis of the Avon Longitudinal Study of Parents and Children (ALSPAC) cohort reported a graded inverse association between physical activity and obesity at age 12[11]; an increase of 15 minutes of moderate to vigorous intensity physical activity (MVPA) per day was associated with 10% lower fat mass in girls and 12% lower in boys[12]. However, the authors used an ALSPAC-specific definition of MVPA[13] and reported an average MVPA of 20 minutes per day while the majority of current studies use the Evenson cut-points[14] which a comparison study reported was the reliable threshold[15] for young people. This difference is important as other studies that use Evenson cut-points typically report MVPA in the range of 50 to 70 minutes per day[16-18] and as such it is important to have data from ALSPAC that have been processed using the Evenson cut-points to facilitate international comparisons.

The overall objective of this paper is to examine the physical activity of adolescents with three of the most common long-term conditions and if these associations change as young people move through adolescence, using objective assessments of physical activity. This evidence is needed to identify whether there are important differences between children with different long-term conditions. The specific aims were: 1) to compare mean time spent sedentary and engaged in MVPA for adolescents with asthma, eczema and obesity at ages 12, 14 and 16; and 2) to examine prospective associations between sedentary and MVPA time from age 12 to 16 for those with and without these conditions.

METHODS

The Avon Longitudinal Study of Parents and Children (ALSPAC) is a birth cohort study based in the former county of Avon (UK)[19-21]. A total of 15247 pregnant women with

For peer review only - http://bmjopen.bmj.com/site/about/guidelines.xhtml

expected delivery dates between April 1991 and December 1992 were recruited, with 14701 children alive at 1 year of age. Ethical approval for the study was obtained from the ALSPAC Ethics and Law Committee and the Local Research Ethics Committees, and written informed consent was obtained for all mothers, with parents providing written informed consent for young people's participation. Please note that the study website contains details of all the data that is available through a fully searchable data dictionary (http://www.bris.ac.uk/alspac/researchers/data-access/data-dictionary/).

Accelerometer Data

Adolescents wore an Actigraph AM7164 (Actigraph LLC, Fort Walton Beach, FL, USA) accelerometer on their hip for seven consecutive days. The first accelerometer measurement was taken at an average age of 11 years and 9 months in 2003-4, with subsequent measurements at ages 13 years and 10 months, and at 15 years and 6 months. Accelerometer data were processed using Kinesoft (v3.3.75; Kinesoft, Saskatchewan, Canada) and analysis was restricted to those who provided at least three days of valid data. A valid day was defined as at least 500 minutes of data, after excluding intervals of ≥60 minutes of zero counts allowing up to two minutes of interruptions. The average number of MVPA (≥2296 counts per minute (CPM)) and sedentary minutes per day (<100 CPM) were derived for each adolescent based on the Evenson thresholds[14]. Accelerometer data with over 11715 CPM, fewer than 10 sedentary minutes or zero MVPA were considered erroneous and excluded from the analysis (16, 41 and 21 respondents at ages 12, 14 and 16 respectively)[22].

Exposures

Height and weight were measured at the same time as the accelerometer data in a clinic setting by trained fieldworkers. Body mass index (BMI) was used to create age- and sex-specific overweight and obesity indicators using 85th and 95th percentiles based on UK BMI

For peer review only - http://bmjopen.bmj.com/site/about/guidelines.xhtml

reference charts[23]. Throughout this paper the overweight category comprises those participants who were overweight but not the participants who were obese, who are defined as obese throughout. The mother was asked 'Has a doctor ever told you that your child has asthma?' at ages 8, 11 and 14, and 'Has a doctor ever told you that your child has eczema?' at ages 11 and 14. These were combined to form lifetime doctor-diagnosed asthma and eczema prevalence: 'has asthma ever been reported as diagnosed by a doctor?' and 'has eczema ever been reported as diagnosed by a doctor?', assessed by age 11 and 14. As these ages do not correspond to accelerometer collection, current asthma/eczema assessments were unavailable.

Confounders

The child's sex as recorded at birth and the age of the child at the accelerometer data collection were included as confounders. The mother's highest education variable was created by combining from data at 32w gestation and age 8 to provide the highest education recorded in the dataset. Household social class was calculated as the highest occupational social class reported by mother or partner from data at 18-weeks' gestation (both) and ages 4 (mother) and 8 (partner). Puberty was assessed from yearly questionnaires. For girls, an indicator of onset of menarche was used. For boys, Tanner stages based on pubic hair was used, as self-report data on Tanner stages for genitals has been found to be unreliable[24].

Patient and Public Involvement

The concept for this paper was informed by previous research with children with asthma and their parents who expressed challenges in relation to being physically active [25]. As this was a secondary analysis of data that were collected 10 years ago participants were not involved in the design of the specific study or study recruitment which were conducted as part of the ALSPAC cohort. Results will be shared with ALSPAC participants via the study website.

For peer review only - http://bmjopen.bmj.com/site/about/guidelines.xhtml

Statistical Analysis

Cross-sectional data were analysed at three timepoints corresponding to accelerometer data at average ages of 12, 14 and 16. We included any child with at least 3 valid days of accelerometer data. Obesity and overweight were calculated at the same timepoints. Asthma and eczema diagnoses did not correspond to the same timepoints, and so were taken from the closest prior measurement, to record lifetime diagnosis up to the point of the accelerometer measurement. Mother's highest education and household social class were calculated as described above and the same value used for all analyses. Cross-sectional analysis was based on 5735, 4078 and 2198 cases for ages 12, 14 and 16 respectively. For the longitudinal analyses we combined data from all three timepoints, resulting in 6473 cases with data for at least one timepoint, and 1619 (25%) at all three.

Multiple imputation was used to account for missing data. For the cross-sectional analyses data were imputed for each dataset separately. For the longitudinal analysis, data were imputed if the child had accelerometer data for that timepoint, but not between timepoints, although data from other timepoints was used to impute missing data. Multiple imputation methods were used to create 20 imputed datasets at each timepoint for boys and girls separately, using 20 cycles of regression switching and combined regression coefficients across datasets using Rubin's rules[26]. All exposures, outcomes and confounders were included.

Cross-sectional Analyses

For each age, characteristics were described separately for all adolescents, those with asthma, eczema, who were overweight or obese, and for adolescents with none of the conditions. Average minutes of MVPA and sedentary time were compared between those with long-term conditions and those without using t-tests. Regression models were fitted for MVPA and

For peer review only - http://bmjopen.bmj.com/site/about/guidelines.xhtml

sedentary minutes for all adolescents, and for boys and girls separately. Models include the asthma, eczema, overweight and obese indicators, and child age, sex, mother's education and household social class as confounders. For comparative purposes, minimally adjusted models which adjusted for just child age and sex were also run and are shown in supplementary tables.

Longitudinal Analysis

Random effect models were fitted to identify changes in physical activity measures over time between the long-term conditions, accounting for individual variability in measurements. As the cross-sectional analyses suggest different patterns among boys and girls, all models were run separately by sex. We included indicators for asthma, eczema, obesity and overweight, and age at time of clinic to capture linear changes over time. All models were adjusted for mother's highest education and household social class. In this model, the constant term captures the average physical activity of a healthy child (i.e. with none of the long-term conditions) at age 12 for average confounder values. The age coefficient estimates a linear change in physical activity (or sedentary time) over time, which is the same for all long-term conditions, and additional terms for each health condition reflect any difference in the baseline activity.

A model was also fitted to assess if there were interactions between the long-term conditions to assess whether there was any additional effect associated with having multiple conditions. Finally, a model which allowed the change in physical activity over time to depend on health condition; so, for example, MVPA for an adolescent with asthma might decrease at a different rate between ages 12 and 16 than for an adolescent without was run. As self-reported pubertal status is susceptible to error[27] and pubertal status could be linked to

For peer review only - http://bmjopen.bmj.com/site/about/guidelines.xhtml

obesity[28], pubertal status was considered in a secondary sensitivity analysis. All analyses were performed in Stata version 15.0[29].

RESULTS

Supplementary Table S1 summarises the characteristics of the imputed data, with summaries for observed data reported in Supplementary Table S2. Missing data ranged from <1% (obesity) to 15% (eczema). Between 24% and 27% of adolescents had ever been diagnosed with asthma, between 21% and 27% had ever been diagnosed with eczema, and between 13% and 15% were obese. Between 13% and 15% had more than one condition, with the most common combination being asthma and eczema (between 7% and 9% of adolescents). The combinations at each age are summarised graphically in Supplementary Figure S1. The average minutes of MVPA per day decreased from 57 mins at age 12 to 47 mins at age 16, with average sedentary minutes increasing from 354 mins to 475 mins. Supplementary Figure S2 illustrates how MVPA and sedentary time changes with age among adolescents with the different conditions.

Cross-sectional Analyses

Supplementary Table S3 summarises the difference in MVPA and sedentary time for adolescents with asthma, eczema, overweight and obese conditions, compared to those without. Tables 1 and 2 present the regression models for MVPA and sedentary time respectively, adjusting for age, sex, mother's education and household social class, and report the difference between an adolescent with and without each health condition. These models include all conditions, including combinations; differences are additive if an adolescent has multiple conditions. A minimally adjusted model (Supplementary Table S4), adjusting only

For peer review only - http://bmjopen.bmj.com/site/about/guidelines.xhtml

for child age and sex, shows very little difference to the coefficients reported in the full model.

The largest and most consistent differences are for adolescents who are overweight or obese. MVPA was 2.3-7.5 minutes lower among overweight adolescents, and 5.3- 8.9 minutes lower among obese adolescents. Similarly, sedentary time was 8.6-13.8 minutes higher among overweight adolescents, and 14.1-22.1 minutes higher among obese adolescents. These associations tend to be slightly stronger in boys than girls. Patterns for adolescents with asthma and eczema are weaker and less consistent. Associations between MVPA and asthma were inconclusive, with a lower MVPA (2.6 mins) among asthma sufferers at age 16, but no other ages, and no differences when examining boys and girl separately. There was some association between asthma and sedentary time, with lower sedentary time at ages 12 and 14 (by 5.1 mins and 10.8 mins respectively), although evidence is inconclusive when

For peer review only

For peer review only - http://bmjopen.bmj.com/site/about/guidelines.xhtml

Table 1: Cross-sectional regression models for MVPA at average age 12, 14 and 16

	Age 12 (n=5735)			Age 14 (n=4078)			Age 16 (n=2198)		
	Diff[2] (mins)	95 % CI	p-value	Diff[2] (mins)	95 % CI	p-value	Diff[2] (mins)	95 % CI	p-value
**All[1]**									
Asthma	-1.47	(-3.19, 0.25)	0.094	-0.05	(-2.05, 1.95)	0.960	-2.62	(-5.16, -0.09)	0.043
Eczema	-1.85	(-3.68, -0.01)	0.049	-0.62	(-2.69, 1.44)	0.554	0.74	(-1.75, 3.24)	0.559
Overweight	-7.54	(-9.59, -5.48)	<0.0005	-3.12	(-5.53, -0.72)	0.011	-2.27	(-5.55, 1.01)	0.175
Obese	-8.94	(-10.92, -6.96)	<0.0005	-7.45	(-9.87, -5.04)	<0.0005	-5.29	(-8.50, -2.07)	0.001
**Boys[1]**									
Asthma	-2.03	(-4.76, 0.70)	0.144	0.47	(-2.71, 3.65)	0.773	-3.22	(-7.42, 0.98)	0.133
Eczema	-3.52	(-6.70, -0.34)	0.030	0.05	(-3.51, 3.61)	0.977	2.28	(-2.16, 6.71)	0.314
Overweight	-11.39	(-14.83, -7.95)	<0.0005	-4.47	(-8.54, -0.40)	0.031	-5.22	(-10.95, 0.50)	0.074
Obese	-12.52	(-15.77, -9.26)	<0.0005	-10.24	(-14.13, -6.35)	<0.0005	-9.42	(-14.99, -3.85)	0.001
**Girls[1]**									
Asthma	-1.01	(-3.09, 1.06)	0.339	-0.71	(-3.16, 1.75)	0.573	-2.48	(-5.52, 0.56)	0.110
Eczema	-0.49	(-2.52, 1.55)	0.639	-1.17	(-3.75, 1.41)	0.372	-0.34	(-3.16, 2.49)	0.816
Overweight	-4.07	(-6.44, -1.70)	0.001	-1.95	(-4.79, 0.88)	0.177	0.19	(-3.59, 3.96)	0.923
Obese	-5.39	(-7.70, -3.07)	<0.0005	-4.52	(-7.49, -1.55)	0.003	-1.88	(-5.59, 1.84)	0.321

[1] Models include all conditions, and are adjusted for child age, sex, mother's education and household social class as confounders.
[2] Difference in minutes of MVPA between a child with and without the condition, with all other variables the same.

For peer review only

Table 2: Cross-sectional regression models for sedentary time at average age 12, 14 and 16

	Age 12 (n=5735)			Age 14 (n=4078)			Age 16 (n=2198)		
	Diff[2] (mins)	95 % CI	p-value	Diff[2] (mins)	95 % CI	p-value	Diff[2] (mins)	95 % CI	p-value
**All[1]**									
Asthma	-5.14	(-9.80, -0.49)	0.030	-10.77	(-16.90, -4.65)	0.001	0.89	(-7.30, 9.09)	0.831
Eczema	0.76	(-4.25, 5.78)	0.765	4.02	(-2.55, 10.59)	0.230	0.95	(-7.09, 9.00)	0.816
Overweight	13.82	(8.334, 19.30)	<0.0005	13.53	(6.17, 20.88)	<0.0005	8.63	(-1.96, 19.22)	0.110
Obese	14.86	(9.59, 20.13)	<0.0005	22.06	(14.68, 29.43)	<0.0005	14.11	(3.75, 24.48)	0.008
**Boys[1]**									
Asthma	-5.86	(-12.33, 0.60)	0.075	-8.17	(-17.15, 0.81)	0.074	3.47	(-9.06, 16.01)	0.587
Eczema	2.64	(-4.63, 9.90)	0.476	2.80	(-7.19, 12.80)	0.582	-2.30	(-15.58, 10.98)	0.734
Overweight	15.07	(7.03, 23.12)	<0.0005	9.49	(-1.78, 20.75)	0.099	16.70	(-0.21, 33.62)	0.053
Obese	13.44	(5.82, 21.05)	0.001	29.28	(18.49, 40.06)	<0.0005	21.78	(5.32, 38.25)	0.010
**Girls[1]**									
Asthma	-4.41	(-11.00, 2.19)	0.190	-13.26	(-21.81, -4.72)	0.002	-0.69	(-11.54, 10.17)	0.901
Eczema	-0.85	(-7.67, 5.96)	0.806	4.55	(-4.29, 13.40)	0.312	3.33	(-6.97, 13.63)	0.526
Overweight	12.69	(5.19, 20.19)	0.001	17.43	(7.74, 27.12)	<0.0005	1.87	(-11.66, 15.41)	0.782
Obese	15.89	(8.55, 23.23)	<0.0005	14.44	(4.30, 24.58)	0.005	7.63	(-5.70, 20.96)	0.262

[1] Models include all conditions, and are adjusted for child age, sex, mother's education and household social class as confounders.

[2] Difference in minutes of sedentary time between a child with and without the condition.

For peer review only

considering boys and girls separately. There was very little association between eczema and either MVPA or sedentary time. We saw a slightly lower MVPA (3.5 mins) among boys at age 12, but no other ages, and no associations with sedentary time. It was not possible to explore associations with combinations of long-term conditions, due to small numbers (<100) for some combinations. However, there was no evidence of any additional association with having multiple conditions. So, for example, the difference between obese children with asthma and obese children without asthma is the same as the difference between non-obese children with asthma and non-obese children without.

Longitudinal Analysis

Table 3 summarizes the longitudinal models for MVPA and sedentary time, for boys and girls separately. The baseline represents a typical child at age 12 with the most common mother's highest education (O level – exams at age 16), and household class (Managerial & Technical). These coefficients are additive, so, for example, the average number of minutes of MVPA for a 12-year-old obese boy is estimated as 69.7-11.1 = 58.6 mins. Figure 1 plots the estimated average MVPA time and sedentary time for the different long-term conditions by age, for boys and girls.

The largest association was between physical activity and obesity, especially among boys. Asthma and eczema showed much weaker associations. A typical boy with no long-term conditions engaged in an average of 72.8 mins of MVPA at age 12, declining at a rate of 3.1 mins/year with age. They spent an average of 318.5 mins in sedentary time at age 12, increasing at a rate of 28.1 mins/year. Girls' average MVPA was lower at 49.3 mins at age 12, declining at a rate of 1.8 mins/year, while sedentary time was higher, with an average of 333.8 mins at age 12, increasing at a rate of 32.0 mins/year. We found no association between

For peer review only - http://bmjopen.bmj.com/site/about/guidelines.xhtml

Table 3: Comparison of longitudinal models for average minutes of MVPA and sedentary time per day

	Boys (2510 boys, 4847 measurements)			Girls (2744 girls, 5481 measurements)		
	**Estimate**	**95% CI**	**P value**	**Estimate**	**95% CI**	**P value**
**Average MVPA minutes per day**						
Baseline [1] (mins)	69.68	(67.64, 71.71)	<0.0005	47.52	(46.13, 48.90)	<0.0005
Change with age (mins/year)	-3.09	(-3.578, -2.61)	<0.0005	-1.78	(-2.10, -1.45)	<0.0005
Asthma[2] (mins)	-1.24	(-3.35, 0.87)	0.249	-1.19	(-2.81, 0.43)	0.150
Eczema[2] (mins)	-1.22	(-3.62. 1.17)	0.316	-0.87	(-2.52, 0.79)	0.305
Overweight[2] (mins)	-8.05	(-10.46, -5.63)	<0.0005	-2.33	(-3.99, -0.68)	0.006
Obese[2] (mins)	-11.12	(-13.57, -8.68)	<0.0005	-5.01	(-6.78, -3.25)	<0.0005
**Average sedentary minutes per day**						
Baseline [1] (mins)	346.64	(341.19, 352.08)	<0.0005	365.73	(31.05, 370.41)	<0.0005
Change with age (mins/year)	28.10	(26.94, 29.26)	<0.0005	31.97	(30.92, 33.03)	<0.0005
Asthma[2] (mins)	-4.06	(-9.84, 1.73)	0.169	-6.45	(-11.92, -0.98)	0.021
Eczema[2] (mins)	2.16	(-4.17, 8.49)	0.504	1.98	(-3.54, 7.50)	0.482
Overweight[2] (mins)	14.51	(8.38, 20.63)	<0.0005	12.63	(7.14, 18.12)	<0.0005
Obese[2] (mins)	17.37	(10.97, 23.78)	<0.0005	15.19	(9.28, 21.09)	<0.0005

[1] Baseline represents the average number of minutes for a healthy individual at age 11 with mother's highest education of O level (exam at age 16) and household social class of Managerial & Technical (II)

[2] Change from baseline if child has this health condition

For peer review only

asthma and either MVPA or sedentary time for boys. Among girls, there was no association between MVPA and asthma, but asthma sufferers spent 6.5 fewer minutes in sedentary time than non-sufferers. We found no association between eczema and either MVPA or sedentary time for boys or girls.

Overweight and obese adolescents had lower MVPA and higher sedentary time. Associations are stronger for boys than for girls, and stronger for obesity than overweight. Being overweight is associated with a decrease in MVPA of 8.1 mins and an increase in sedentary time of 14.5 mins for boys, compared to a decrease of 2.3 mins in MVPA and an increase of 12.6 mins in sedentary time for girls. Obesity is associated with a decrease in MVPA of 11.1 mins and an increase in sedentary time of 17.4 mins among boys, and a decrease in MVPA of 5.0 mins and an increase in sedentary time of 15.2 mins among girls.

There was no evidence of any additional effect associated with having combinations of multiple long-term conditions, via interaction terms. This suggests that any associations between physical activity and long-term conditions may be additive, although low prevalence of multiple conditions in this sample mean that we lacked statistical power to explore this fully. There was no evidence that the rate of change in physical activity over time differed between health condition. Including puberty in the longitudinal model (results not shown) reduced the strength of association with obesity and being overweight slightly but had no effect on associations with asthma or eczema.

DISCUSSION

In this paper we found no strong evidence of differences in the mean minutes of MPVA or sedentary time per day between adolescents with asthma or eczema and those without these

For peer review only - http://bmjopen.bmj.com/site/about/guidelines.xhtml

conditions in the cross-sectional models. We found a small association between asthma and fewer minutes of sedentary time among girls, but no associations for MVPA or eczema. Results suggest that adolescents who are overweight or obese engage in lower levels of physical activity at ages 12, 14 and 16, with stronger associations for boys than girls. These findings were confirmed in the longitudinal models which showed lower levels of MVPA and higher sedentary time with increasing levels of obesity. Specifically, obese boys engage in 11.1 fewer minutes of MVPA and 17.4 more sedentary minutes compared to a boy of similar age and demographics, while obese girls engage in 5.0 fewer minutes of MVPA and 15.2 more sedentary minutes than their non-obese counterparts. This finding is consistent with a previous Mendelian randomization analysis which suggested a likely bi-directional association between physical activity and adiposity among young people[30].

The lack of an association between physical activity, sedentary time and asthma challenges a body of research which has reported that children with asthma are less active than their peers. However, previous literature has relied on relatively small cross-sectional studies using self-reported assessments of physical activity and asthma[8 31-33]. The current analysis uses a population-based rather than clinical cohort and therefore contains a lower proportion of children with moderate to severe asthma. Also, because the study combines data measured at different times, we report lifetime asthma prevalence rather than current asthma. Therefore, caution should be exercised in any direct comparison with other studies. Low physical activity and fitness have been suggested as risk factors for asthma onset[34] and exercise has been shown to improve markers of asthma control[35], which appear inconsistent with our findings. Concerns that exercise-induced bronchoconstriction might limit physical activity in children with controlled asthma appear to be ill-founded[36]. Our results therefore provide reassurance that adolescents with asthma in a general population take part in similar levels of

For peer review only - http://bmjopen.bmj.com/site/about/guidelines.xhtml

physical activity to their peers and there is no need for condition-specific behaviour change programs.

A previous Mendelian randomisation study from the ALSPAC cohort at age 7 identified obesity as a risk factor for asthma[37]. This is consistent with our results. We observed a higher prevalence of obesity amongst asthma sufferers (20%, 16% and 17% at ages 12, 14 and 16) than those without asthma (14%, 12% and 12% respectively). Furthermore, obesity with asthma has been implicated as a risk for more frequent asthma exacerbations[38], condition severity and a reduced response to therapy[39]. Some small studies have suggested that weight loss may aid asthma management in children and adults[40-42]. These studies and the consistent reporting of associations between asthma, obesity prevalence and condition morbidity supported by this study suggest that larger and longer weight management trials in obese individuals with asthma are warranted.

Previous evidence of any difference in the physical activity patterns of children with and without eczema was unclear[9]. There is some evidence of an association in adults[43], and possibly an association with severe eczema in children[44]. There may also be a link between eczema and obesity in North America and Asia, although there is no evidence of an association in Europe[45]. Our analyses found no differences in accelerometer-measured physical activity for those with lifetime-diagnosed eczema of any severity, which is encouraging given the psychosocial impact of even mild eczema. While parents may restrict activities such as swimming because of concerns about the effects on the child's skin[46], our findings suggest that the presence of eczema does not overall negatively impact physical activity in this group and therefore there is no need for eczema-specific physical activity programs.

For peer review only - http://bmjopen.bmj.com/site/about/guidelines.xhtml

Strengths and limitations

The main strength of this study is the objective assessment of physical activity at ages, 12, 14 and 16 and multiple assessments of physical conditions on a large cohort of adolescents. Although only 25% participants had data at all three timepoints, 61% had data for at least two, making the longitudinal analysis reasonably robust, although the study was not powered to test for interactions between conditions. We used multiple imputation to minimize the amount of missing data but recognize that there are limitations of this approach, including the underlying assumption that data are missing at random[47 48].

The study combines data measured at different times. Specifically, the asthma and eczema assessments do not exactly coincide with the physical activity data and so we were unable to include current asthma/eczema indicators. We also have no asthma/eczema information between 11 and 14 and so cannot determine, for example, if asthma precedes obesity. We have not adjusted for smoking we may have impacted on other associations. It is also important to highlight that due to the small number of cases we were unable to model the interactions between health conditions. Finally, we are unable to differentiate between mild and severe asthma or eczema – more severe cases tend to be more persistent and it may be that persistence is linked to activity. The data were collected approximately a decade ago and it is possible that physical activity patterns and the prevalence of physical conditions, especially obesity, may have changed over this period.

CONCLUSIONS

For peer review only - http://bmjopen.bmj.com/site/about/guidelines.xhtml

Analysis of the ALSPAC population-based cohort has shown that physical activity and sedentary time did not differ for adolescents with asthma or eczema and those without, but obese adolescents engaged in fewer minutes of MVPA and more sedentary time. Findings reinforce the need for strategies to help obese adolescents be more active but suggest no need to develop bespoke physical activity strategies for adolescents with mild asthma or eczema.

ACKNOWLEDGEMENTS

We are extremely grateful to all the families who took part in this study, the midwives for their help in recruiting them, and the whole ALSPAC team, which includes interviewers, computer and laboratory technicians, clerical workers, research scientists, volunteers, managers, receptionists and nurses.

FUNDING

The UK Medical Research Council and Wellcome (Grant ref: 102215/2/13/2) and the University of Bristol provide core support for ALSPAC. This publication is the work of the authors and AJH, ARN, RES and RJ will serve as guarantors for the contents of this paper. A comprehensive list of grants funding is available on the ALSPAC website. Physical activity data collection was supported by the US National Heart, Lung, and Blood Institute (R01 HL071248-01A). RJ is partly supported by the National Institute for Health Research (NIHR) Collaboration for Leadership in Applied Health Research and Care West (CLAHRC West) at University Hospitals Bristol NHS Foundation Trust. The NIHR Biomedical Research Centre at University Hospitals Bristol NHS Foundation Trust and the University of Bristol supports

For peer review only - http://bmjopen.bmj.com/site/about/guidelines.xhtml

ARN and JPH-S. MR is funded by a National Institute for Health Research Post-Doctoral Research Fellowship (PDF-2014-07-013).

COMPETING INTERESTS: None

CONTRIBUTORS

Conception/design: all

Methodology: RJ, RES, MJR, AJH, ARN, JPHS

Data analysis: RES

Drafting: RJ, RES

Revising: all

Final approval: all

DATA SHARING STATEMENT

This is a secondary data analysis based on data from the ALSPAC cohort. The access policy for the ALSPAC data can be found at http://www.bristol.ac.uk/media-library/sites/alspac/documents/researchers/data-access/ALSPAC_Access_Policy.pdf

DISCLAIMER

The views expressed in this publication are those of the author(s) and not necessarily those of the NHS, the National Institute for Health Research or the Department of Health.

For peer review only

For peer review only - http://bmjopen.bmj.com/site/about/guidelines.xhtml

REFERENCES

1. National Statistics. National Child Measurement Programme, England 2016-2017 school year. 2016
2. Flohr C, Mann J. New insights into the epidemiology of childhood atopic dermatitis. *Allergy* 2014;69(1):3-16. doi: 10.1111/all.12270
3. Asthma UK. Asthma facts and statistics 2017 [Available from: https://www.asthma.org.uk/about/media/facts-and-statistics/ accessed 13/03/2017.
4. Reichert FF, Baptista Menezes AM, Wells JC, et al. Physical activity as a predictor of adolescent body fatness: a systematic review. *Sports Med* 2009;39(4):279-94. doi: 10.2165/00007256-200939040-00002
5. Lochte L, Nielsen KG, Petersen PE, et al. Childhood asthma and physical activity: a systematic review with meta-analysis and Graphic Appraisal Tool for Epidemiology assessment. *BMC Pediatr* 2016;16:50. doi: 10.1186/s12887-016-0571-4
6. Cordova-Rivera L, Gibson PG, Gardiner PA, et al. A systematic review of associations of physical activity and sedentary time with asthma outcomes. *J Allergy Clin Immunol Pract* 2018 doi: 10.1016/j.jaip.2018.02.027 [published Online First: 2018/03/07]
7. World Health Organization. Global health risks: mortality and burden of disease attributable to selected major risks: World Health Organization, , 2009.
8. Cassim R, Koplin JJ, Dharmage SC, et al. The difference in amount of physical activity performed by children with and without asthma: A systematic review and meta-analysis. *J Asthma* 2016;53(9):882-92. doi: 10.1080/02770903.2016.1175474
9. Kim A, Silverberg JI. A systematic review of vigorous physical activity in eczema. *Br J Dermatol* 2016;174(3):660-2. doi: 10.1111/bjd.14179
10. Coombs NA, Stamatakis E. Associations between objectively assessed and questionnaire-based sedentary behaviour with BMI-defined obesity among general population children and adolescents living in England. *BMJ open* 2015;5(6):e007172. doi: 10.1136/bmjopen-2014-007172
11. Ness AR, Leary SD, Mattocks C, et al. Objectively Measured Physical Activity and Fat Mass in a Large Cohort of Children. *PLoS Med* 2007;4(3):e97.
12. Riddoch CJ, Leary SD, Ness AR, et al. Prospective associations between objective measures of physical activity and fat mass in 12-14 year old children: the Avon Longitudinal Study of Parents and Children (ALSPAC). *BMJ* 2009;339:b4544. doi: 10.1136/bmj.b4544 [published Online First: 2009/11/28]
13. Mattocks C, Leary S, Ness A, et al. Calibration of an accelerometer during free-living activities in children. *Int J Pediatr Obes* 2007:1-9.
14. Evenson KR, Catellier DJ, Gill K, et al. Calibration of two objective measures of physical activity for children. *J Sports Sci* 2008;26(14):1557-65. doi: 1080/02640410802334196.
15. Trost SG, Loprinzi PD, Moore R, et al. Comparison of Accelerometer Cut-points for Predicting Activity Intensity in Youth. *Med Sci Sports Exerc* 2011;43(7)
16. Cooper AR, Goodman A, Page AS, et al. Objectively measured physical activity and sedentary time in youth: the International children's accelerometry database (ICAD). *Int J Behav Nutr Phys Act* 2015;12:113. doi: 10.1186/s12966-015-0274-5
17. Harding SK, Page AS, Falconer C, et al. Longitudinal changes in sedentary time and physical activity during adolescence. *Int J Behav Nutr Phys Act* 2015;12:44. doi: 10.1186/s12966-015-0204-6
18. Jago R, Solomon-Moore E, Macdonald-Wallis C, et al. Change in children's physical activity and sedentary time between Year 1 and Year 4 of primary school in the B-

For peer review only - http://bmjopen.bmj.com/site/about/guidelines.xhtml

PROACT1V cohort. *Int J Behav Nutr Phys Act* 2017;14(1):33. doi: 10.1186/s12966-017-0492-0
19. Golding J, Pembrey M, Jones R. ALSPAC--the Avon Longitudinal Study of Parents and Children. I. Study methodology. *Paediatr Perinat Epidemiol* 2001;15(1):74-87.
20. Boyd A, Golding J, Macleod J, et al. Cohort Profile: the 'children of the 90s'--the index offspring of the Avon Longitudinal Study of Parents and Children. *Int J Epidemiol* 2013;42(1):111-27. doi: 10.1093/ije/dys064 [published Online First: 2012/04/18]
21. Fraser A, Macdonald-Wallis C, Tilling K, et al. Cohort Profile: The Avon Longitudinal Study of Parents and Children: ALSPAC mothers cohort. *International Journal of Epidemiology* 2013;42:97-110.
22. Adams MA, Johnson WD, Tudor-Locke C. Steps/day translation of the moderate-to-vigorous physical activity guideline for children and adolescents. *Int J Behav Nutr Phys Act* 2013;10:49. doi: 10.1186/1479-5868-10-49 [published Online First: 2013/04/23]
23. Cole TJ, Freeman JV, Preece MA. Body mass index reference curves for the UK, 1990. *Arch Dis Child* 1995;73(1):25-9.
24. Monteilh C, Kieszak S, Flanders WD, et al. Timing of maturation and predictors of Tanner stage transitions in boys enrolled in a contemporary British cohort. *Paediatr Perinat Epidemiol* 2011;25(1):75-87. doi: 10.1111/j.1365-3016.2010.01168.x [published Online First: 2010/12/08]
25. Jago R, Searle A, Henderson AJ, et al. Designing a physical activity intervention for children with asthma: A Qualitative study of the views of health care professionals, parents and children with asthma. *BMJ open* 2017;7(e014020):1-8.
26. Rubin DB. Multiple imputation after 18+ years. *Journal of the American Statistical Association* 1996;91(434):473-789.
27. Monteilha C, Kieszaka S, Flanders WD, et al. Timing of maturation and predictors of Tanner stage transitions in boys enrolled in a contemporary British cohort. *Paediatric and Perinatal Epidemiology* 2011;25:75-87. doi: 10.1111/j.1365-3016.2010.01168.x
28. Kaplowitz PB. Link Between Body Fat and the Timing of Puberty. *Pediatrics* 2008;121(Supplement 3)
29. Stata Statistical Software: Release 15 [program]. College Station, TX: StataCorp LLC, 2017.
30. Richmond RC, Davey Smith G, Ness AR, et al. Assessing causality in the association between child adiposity and physical activity levels: a Mendelian randomization analysis. *PLoS Med* 2014;11(3):e1001618. doi: 10.1371/journal.pmed.1001618 [published Online First: 2014/03/20]
31. Glazebrook C, McPherson AC, Macdonald IA, et al. Asthma as a barrier to children's physical activity: implications for body mass index and mental health. *Pediatrics* 2006;118(6):2443-9. doi: 10.1542/peds.2006-1846
32. Lam KM, Yang YH, Wang LC, et al. Physical Activity in School-Aged Children with Asthma in an Urban City of Taiwan. *Pediatr Neonatol* 2016;57(4):333-7. doi: 10.1016/j.pedneo.2015.05.003
33. Leinaar E, Alamian A, Wang L. A systematic review of the relationship between asthma, overweight, and the effects of physical activity in youth. *Ann Epidemiol* 2016;26(7):504-10 e6. doi: 10.1016/j.annepidem.2016.06.002 [published Online First: 2016/07/28]
34. Rasmussen F, Lambrechtsen J, Siersted HC, et al. Low physical fitness in childhood is associated with the development of asthma in young adulthood: the Odense schoolchild study. *The European respiratory journal : official journal of the*

For peer review only

For peer review only - http://bmjopen.bmj.com/site/about/guidelines.xhtml

European Society for Clinical Respiratory Physiology 2000;16(5):866-70. [published Online First: 2001/01/12]
35. Lucas SR, Platts-Mills TA. Physical activity and exercise in asthma: relevance to etiology and treatment. *J Allergy Clin Immunol* 2005;115(5):928-34. doi: 10.1016/j.jaci.2005.01.033 [published Online First: 2005/05/04]
36. Orenstein DM. Pulmonary problems and management concerns in youth sports. *Pediatr Clin North Am* 2002;49(4):709-21, v-vi. [published Online First: 2002/09/26]
37. Granell R, Henderson AJ, Evans DM, et al. Effects of BMI, fat mass, and lean mass on asthma in childhood: a Mendelian randomization study. *PLoS Med* 2014;11(7):e1001669. doi: 10.1371/journal.pmed.1001669
38. Ahmadizar F, Vijverberg SJ, Arets HG, et al. Childhood obesity in relation to poor asthma control and exacerbation: a meta-analysis. *The European respiratory journal : official journal of the European Society for Clinical Respiratory Physiology* 2016;48(4):1063-73. doi: 10.1183/13993003.00766-2016 [published Online First: 2016/09/03]
39. Forno E, Celedon JC. The effect of obesity, weight gain, and weight loss on asthma inception and control. *Curr Opin Allergy Clin Immunol* 2017;17(2):123-30. doi: 10.1097/ACI.0000000000000339 [published Online First: 2016/12/29]
40. Willeboordse M, van de Kant KDG, Tan FE, et al. A Multifactorial Weight Reduction Programme for Children with Overweight and Asthma: A Randomized Controlled Trial. *PLoS One* 2016;11(6):e0157158. doi: 10.1371/journal.pone.0157158 [published Online First: 2016/06/15]
41. Luna-Pech JA, Torres-Mendoza BM, Luna-Pech JA, et al. Normocaloric diet improves asthma-related quality of life in obese pubertal adolescents. *Int Arch Allergy Immunol* 2014;163(4):252-8. doi: 10.1159/000360398 [published Online First: 2014/04/10]
42. Adeniyi FB, Young T. Weight loss interventions for chronic asthma. *Cochrane Database Syst Rev* 2012(7):CD009339. doi: 10.1002/14651858.CD009339.pub2 [published Online First: 2012/07/13]
43. Silverberg JI, Song J, Pinto D, et al. Atopic Dermatitis Is Associated with Less Physical Activity in US Adults. *J Invest Dermatol* 2016;136(8):1714-6.
44. Strom MA, Silverberg JI. Associations of Physical Activity and Sedentary Behavior with Atopic Disease in United States Children. *J Pediatr* 2016;174:247-53.e3.
45. Zhang A, Silverberg JI. Association of atopic dermatitis with being overweight and obese: a systematic review and metaanalysis. *J Am Acad Dermatol* 2015;72(4):606-16. doi: 10.1016/j.jaad.2014.12.013
46. Santer M, Burgess H, Yardley Y, et al. Experiences of carers managing childhood eczema and their views on its treatment: a qualitative study. *Br J Gen Pract* 2012;62(597):e261-e26.
47. Sterne JA, White IR, Carlin JB, et al. Multiple imputation for missing data in epidemiological and clinical research: potential and pitfalls. *BMJ* 2009;338:b2393. doi: 10.1136/bmj.b2393
48. White IR, Royston P, Wood AM. Multiple imputation using chained equations: Issues and guidance for practice. *Stat Med* 2011;30(4):377-99. doi: 10.1002/sim.4067

For peer review only

For peer review only - http://bmjopen.bmj.com/site/about/guidelines.xhtml

FIGURE LEGENDS AND KEYS

Figure 1: Fitted models for average minutes of MVPA (left) and sedentary time (right) for different long-term conditions and by sex.

Supplementary Figures

Figure S1: Observed average minutes of MVPA (left) and sedentary time (right) for different long-term conditions and by sex.

Figure S2: Venn diagrams of combinations of multiple conditions at ages 12, 14 and 16.

For peer review only

For peer review only

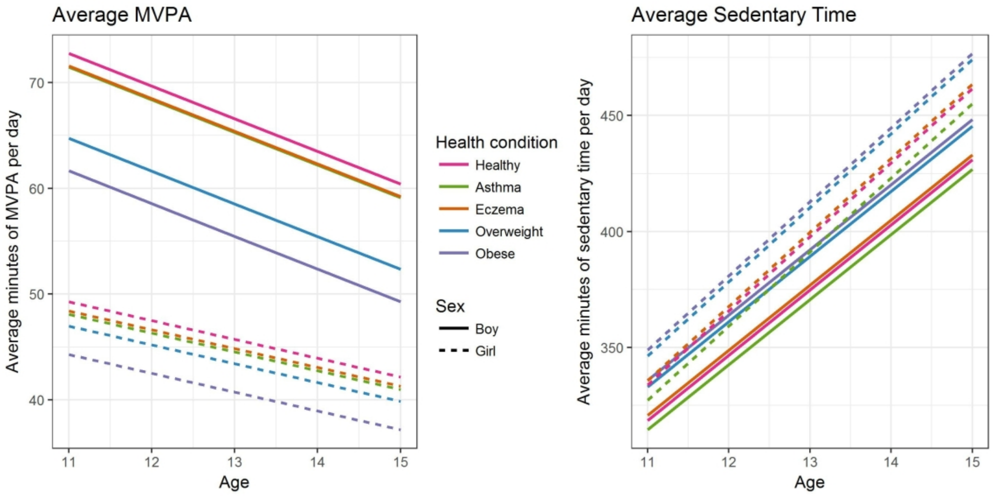

Figure 1: Estimated average MVPA and sedentary time for different health conditions by age for boys and girls

229x114mm (300 x 300 DPI)

SUPPLEMENTARY MATERIAL

Russell Jago, Ruth E. Salway, Andy R Ness, Julian P Hamilton-Shield, Matthew J Ridd, A John Henderson, *Associations between Physical Activity and Asthma, Eczema and Obesity in children aged 12-16*

List of Supplementary Tables

Table S1: Characteristics of imputed data

Table S2: Characteristics of observed data and missing values

Table S3: MVPA and sedentary time by health condition at each time point: unadjusted for confounders or other conditions

Table S4: Cross-sectional regression models for MVPA at average age 12, 14 and 16: minimally adjusted

List of Supplementary Figures

Figure S1: Venn diagrams of combinations of multiple conditions at ages 12, 14 and 16.

Figure S2: Observed average minutes of MVPA (left) and sedentary time (right) for different health conditions and by sex.

For peer review only

Table S1: Characteristics of imputed data

		Age 12 (n=5735)	Age 14 (n=4078)	Age 16 (n=2198)
		Mean (sd)	Mean (sd)	Mean (sd)
MVPA		57 (29)	52 (27)	47 (27)
Sedentary time		354 (73)	415 (81)	475 (84)
		%	%	%
**Condition**				
Asthma		24%	23%	27%
Eczema		21%	21%	27%
Overweight		14%	13%	13%
Obese		15%	13%	12%
**Combinations of conditions**				
None		47%	49%	44%
Asthma only		10%	11%	11%
Eczema only		9%	10%	12%
Obese only		8%	7%	6%
Asthma & Eczema		6%	6%	8%
Asthma & Obese		3%	3%	2%
Eczema & Obese		2%	2%	2%
Asthma, Eczema & Obese		2%	1%	2%
**Confounders**				
Sex (female)		53%	53%	55%
Mother's education:	None/CSE	11%	9%	8%
	Vocational	9%	8%	8%
	O level (exams at 16)	47%	47%	47%
	A level (exams at 18)	15%	16%	15%
	Degree	18%	20%	22%
Social class:	I Professional	13%	14%	16%
	II Manager & Tech	47%	49%	49%
	III Skilled, non-man	26%	25%	23%
	III Skilled, manual	9%	7%	7%
	IV Partly skilled	4%	4%	4%
	V Unskilled	1%	1%	1%

For peer review only

Table S2: Characteristics of observed data and missing values

		Age 12 (n=5735)		Age 14 (n=4078)		Age 16 (n=2198)	
		Mean (sd)	Missing (%)	Mean (sd)	Missing (%)	Mean (sd)	Missing (%)
MVPA		57 (29)	0 (0%)	52 (27)	0 (0%)	47 (27)	0 (0%)
Sedentary time		354 (73)	0 (0%)	415 (81)	0 (0%)	475 (84)	0 (0%)
		%		%		%	
**Condition**							
Asthma		24%	454 (8%)	23%	282 (7%)	27%	74 (3%)
Eczema		21%	842 (15%)	21%	542 (13%)	27%	114 (5%)
Overweight		14%	9 (0.2%)	13%	8 (0.2%)	13%	25 (1%)
Obese		15%	9 (0.2%)	13%	8 (0.2%)	12%	25 (1%)
**Combinations of conditions**							
None		55%	850 (15%)	56%	549 (13%)	51%	137 (6%)
Asthma only		13%		13%		13%	
Eczema only		11%		12%		14%	
Obese only		8%		7%		7%	
Asthma & Eczema		7%		7%		9%	
Asthma & Obese		3%		3%		2%	
Eczema & Obese		2%		2%		2%	
Asthma, Eczema & Obese		2%		1%		2%	
**Confounders**							
Sex (female)		53%	0 (0%)	53%	0 (0%)	55%	0 (0%)
Mother's education:	None/CSE	11%	230 (4%)	9%	136 (4%)	8%	71 (3%)
	Vocational	9%		8%		8%	
	O level (exams at 16)	47%		47%		47%	
	A level (exams at 18)	15%		16%		15%	
	Degree	18%		20%		22%	
Social class:	I Professional	13%	365 (6%)	14%	213 (5%)	16%	118 (5%)
	II Manager & Tech	47%		49%		49%	
	III Skilled, non-man	26%		25%		23%	
	III Skilled, manual	9%		7%		7%	
	IV Partly skilled	4%		4%		4%	
	V Unskilled	1%		1%		1%	

For peer review only

Table S3: MVPA and sedentary time by health condition at each time point: unadjusted for confounders or other conditions

	Age 12 (n=5735)					Age 14 (n=4078)					Age 16 (n=2198)				
	Mean	sd	Diff[1] (mins)	95% CI[1]	p-value[2]	Mean	sd	Diff[1] (mins)	95% CI[1]	p-value[2]	Mean	sd	Diff[1] (mins)	95% CI[1]	p-value[2]
**MVPA**															
Asthma	56	28	-0.47	(-2.29, 1.35)	0.613	53	27	1.25	(-0.79, 3.30)	0.229	46	29	-1.58	(-4.8, 1.02)	0.233
Eczema	54	26	-3.13	(-5.-8, -1.18)	0.002	51	27	-1.44	(-0.358, 0.70)	0.187	46	27	-0.62	(-3.20, 1.96)	0.638
Overweight	52	24	-5.63	(-7.81, -3.44)	<0.0005	50	27	-2.47	(-4.98, 0.03)	0.053	45	28	-1.64	(-5.04, 1.76)	0.344
Obese	51	25	-7.02	(-9.11, -4.94)	<0.0005	46	25	-6.02	(-8.51, -3.52)	<0.0005	42	25	-5.46	(-8.77, -2.15)	0.001
**Sedentary time**															
Asthma	349	72	-6.66	(-11.31, -2.02)	0.005	405	83	-13.15	(-19.31, -7.00)	<0.0005	474	89	-1.44	(-9.60, 6.72)	0.729
Eczema	355	72	1.12	(-3.86, 6.10)	0.660	418	82	4.11	(-2.60, 10.81)	0.229	477	82	2.82	(-5.22, 10.85)	0.492
Overweight	363	71	9.56	(4.04, 15.08)	0.001	424	86	10.61	(3.11, 18.11)	0.006	480	82	5.44	(-5.19, 16.08)	0.316
Obese	362	75	8.85	(3.57, 14.12)	0.001	428	79	15.26	(7.78, 22.73)	<0.0005	485	86	11.02	(0.67, 21.38)	0.037

[1] Diff and 95% CI are the mean difference between children with the condition and children without the condition and associated 95% confidence interval.

[2] P-value refers to t-test comparing those with the condition to those without.

For peer review only

Table S4: Cross-sectional regression models for MVPA at average age 12, 14 and 16: minimally adjusted

	Age 12 (n=5735)			Age 14 (n=4078)			Age 16 (n=2198)		
	Diff[3] (mins)	95 % CI	p-value	Diff[3] (mins)	95 % CI	p-value	Diff[3] (mins)	95 % CI	p-value
**MVPA**									
**All[1]**									
Asthma	-1.34	(-3.06, 0.38)	0.126	0.15	(-1.84, 2.14)	0.884	-2.59	(-5.12, -0.06)	0.045
Eczema	-1.95	(-3.78, -0.12)	0.037	-0.72	(-2.79, 1.35)	0.493	0.67	(-1.84, 3.17)	0.601
Overweight	-7.34	(-9.39, -5.28)	<0.0005	-3.10	(-5.50, -0.69)	0.012	-2.05	(-5.32, 1.22)	0.219
Obese	-8.64	(-10.61, -6.67)	<0.0005	-7.05	(-9.45, -4.65)	<0.0005	-5.15	(-8.35, -1.95)	0.002
**Boys[2]**									
Asthma	-1.95	(-4.68, 0.77)	0.161	0.66	(-2.50, 3.83)	0.680	-2.87	(-7.07, 1.33)	0.180
Eczema	-3.73	(-6.91, -0.54)	0.022	-0.21	(-3.76, 3.35)	0.910	2.03	(-2.42, 6.48)	0.371
Overweight	-11.16	(-14.60, -7.72)	<0.0005	-4.40	(-8.45, -0.35)	0.033	-4.64	(-10.33, 1.06)	0.110
Obese	-12.39	(-15.63, -9.14)	<0.0005	-10.00	(-13.87, -6.14)	<0.0005	-9.08	(-14.62, -3.54)	0.001
**Girls[2]**									
Asthma	-0.89	(-2.97, 1.20)	0.404	-0.56	(-3.01, 1.89)	0.656	-2.67	(-5.71, 0.37)	0.085
Eczema	-0.52	(-2.55, 1.51)	0.616	-1.19	(-3.76, 1.37)	0.361	-0.40	(-3.24, 2.44)	0.780
Overweight	-3.89	(-6.26, -1.53)	0.001	-1.90	(-4.74, 0.93)	0.188	0.07	(-3.70, 3.83)	0.972
Obese	-5.05	(-7.35, -2.76)	<0.0005	-4.11	(-7.06, -1.16)	0.006	-2.03	(-5.73, 1.67)	0.282
**Sedentary time**									
**All[1]**									
Asthma	-6.45	(-11.21, -1.69)	0.008	-12.07	(-18.25, -5.89)	<0.0005	-0.56	(-8.83, 7.72)	0.895
Eczema	1.69	(-3.38, 6.76)	0.513	4.62	(-2.05, 11.30)	0.174	1.81	(-6.30, 9.93)	0.661
Overweight	11.81	(6.26, 17.36)	<0.0005	12.70	(5.28, 20.12)	0.001	6.58	(-4.05, 17.21)	0.225
Obese	11.64	(6.32, 16.97)	<0.0005	18.96	(11.54, 26.37)	<0.0005	11.06	(0.66, 21.46)	0.037
**Boys[2]**									
Asthma	-6.84	(-13.42, -0.25)	0.042	-9.60	(-18.68, -0.52)	0.038	1.25	(-11.48, 13.98)	0.847
Eczema	3.96	(-3.43, 11.35)	0.294	4.63	(-5.47, 14.72)	0.368	-1.48	(-14.91, 11.95)	0.829
Overweight	13.19	(5.03, 21.36)	0.002	8.15	(-3.26, 19.56)	0.161	14.58	(-2.45, 31.61)	0.093
Obese	11.13	(3.443, 18.84)	0.005	26.34	(15.47, 37.22)	<0.0005	18.05	(1.48, 34.62)	0.033
**Girls[2]**									
Asthma	-5.92	(-12.65, 0.81)	0.085	-14.70	(-23.30, -6.10)	0.001	-1.51	(-12.39, 9.37)	0.786
Eczema	-0.25	(-7.14, 6.63)	0.943	4.50	(-4.41, 13.41)	0.321	4.36	(-5.97, 14.69)	0.408
Overweight	10.64	(3.06, 18.22)	0.006	16.48	(6.74, 26.22)	0.001	0.27	(-13.24, 13.77)	0.969
Obese	12.06	(4.68, 19.44)	0.001	11.83	(1.69, 21.98)	0.022	5.58	(-7.71, 18.86)	0.410

[1] Model adjusted for child age and sex. [2] Model adjusted for child age
[3] Coefficients indicate difference in minutes of MVPA/sedentary time between a child with and without the condition.

For peer review only

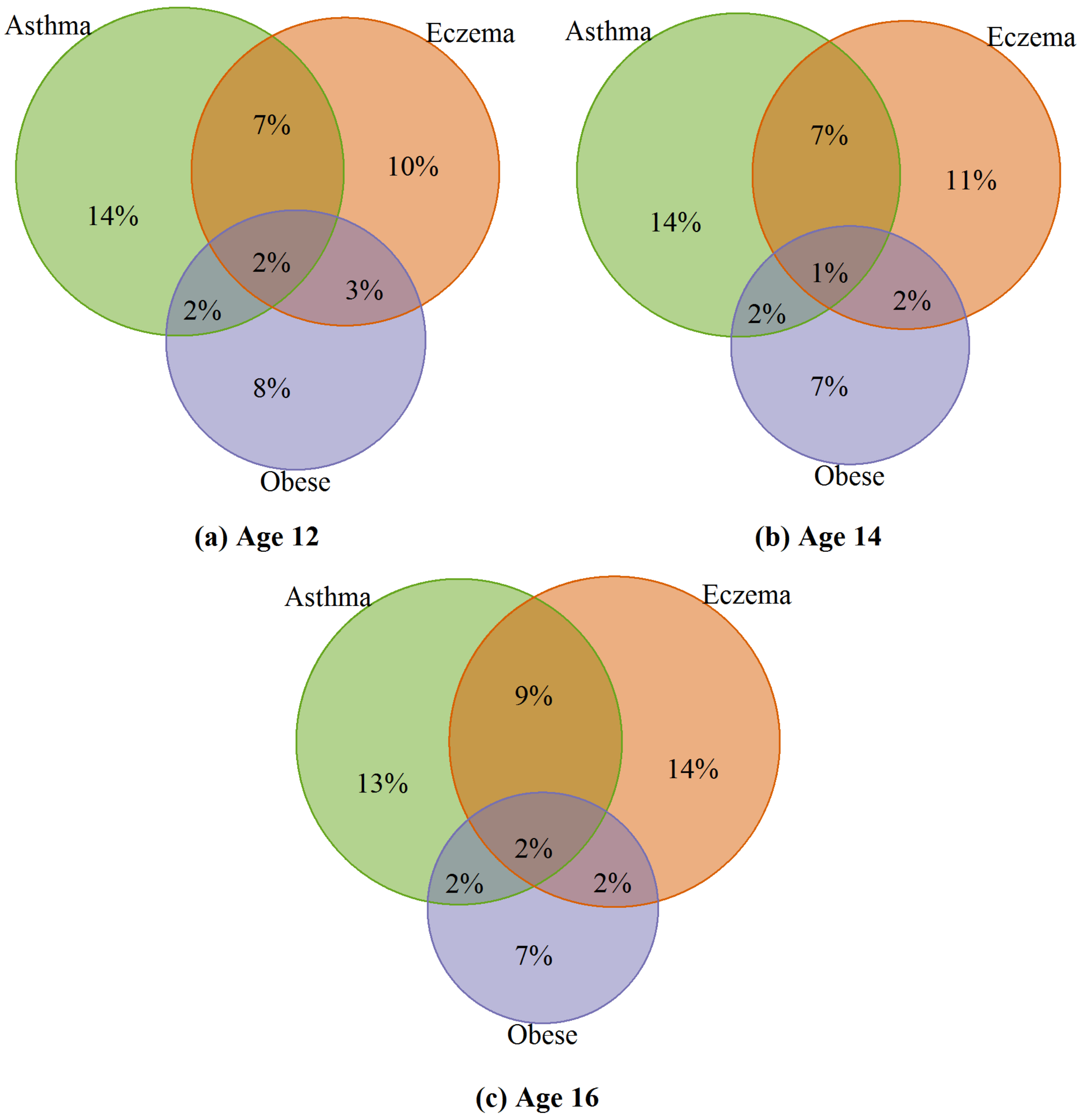

Figure S1: Venn diagrams of combinations of multiple conditions at ages 12, 14 and 16.

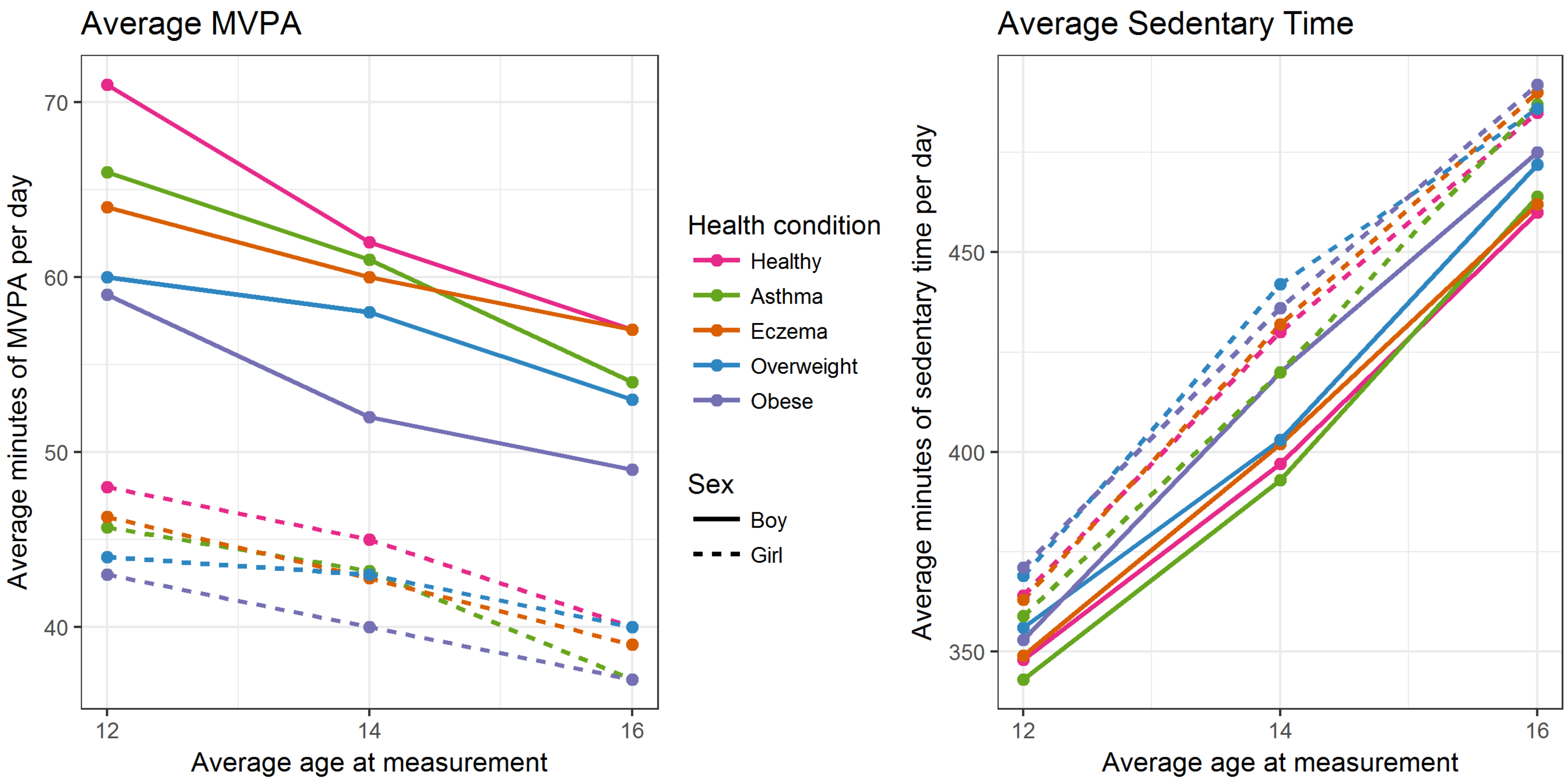

Figure S2: Observed average minutes of MVPA (left) and sedentary time (right) for different health conditions and by sex.

STROBE 2007 (v4) Statement—Checklist of items that should be included in reports of *cohort studies*

Section/Topic	Item #	Recommendation	Reported on page #
**Title and abstract**	1	(*a*) Indicate the study's design with a commonly used term in the title or the abstract	Title page
		(*b*) Provide in the abstract an informative and balanced summary of what was done and what was found	2-3
**Introduction**			
Background/rationale	2	Explain the scientific background and rationale for the investigation being reported	5-6
Objectives	3	State specific objectives, including any prespecified hypotheses	6
**Methods**			
Study design	4	Present key elements of study design early in the paper	6-7
Setting	5	Describe the setting, locations, and relevant dates, including periods of recruitment, exposure, follow-up, and data collection	6-7
Participants	6	(*a*) Give the eligibility criteria, and the sources and methods of selection of participants. Describe methods of follow-up	6-7
		(*b*) For matched studies, give matching criteria and number of exposed and unexposed	N/A
Variables	7	Clearly define all outcomes, exposures, predictors, potential confounders, and effect modifiers. Give diagnostic criteria, if applicable	7-8
Data sources/ measurement	8*	For each variable of interest, give sources of data and details of methods of assessment (measurement). Describe comparability of assessment methods if there is more than one group	7-8
Bias	9	Describe any efforts to address potential sources of bias	8-9
Study size	10	Explain how the study size was arrived at	8
Quantitative variables	11	Explain how quantitative variables were handled in the analyses. If applicable, describe which groupings were chosen and why	9-10
Statistical methods	12	(*a*) Describe all statistical methods, including those used to control for confounding	9-10
		(*b*) Describe any methods used to examine subgroups and interactions	9-10
		(*c*) Explain how missing data were addressed	9
		(*d*) If applicable, explain how loss to follow-up was addressed	n/a
		(*e*) Describe any sensitivity analyses	10
**Results**			

For peer review only

Participants	13*	(a) Report numbers of individuals at each stage of study—eg numbers potentially eligible, examined for eligibility, confirmed eligible, included in the study, completing follow-up, and analysed	8, 10
		(b) Give reasons for non-participation at each stage	n/a
		(c) Consider use of a flow diagram	
Descriptive data	14*	(a) Give characteristics of study participants (eg demographic, clinical, social) and information on exposures and potential confounders	10-11
		(b) Indicate number of participants with missing data for each variable of interest	10
		(c) Summarise follow-up time (eg, average and total amount)	n/a
Outcome data	15*	Report numbers of outcome events or summary measures over time	10
Main results	16	(*a*) Give unadjusted estimates and, if applicable, confounder-adjusted estimates and their precision (eg, 95% confidence interval). Make clear which confounders were adjusted for and why they were included	11-15
		(*b*) Report category boundaries when continuous variables were categorized	n/a
		(*c*) If relevant, consider translating estimates of relative risk into absolute risk for a meaningful time period	n/a
Other analyses	17	Report other analyses done—eg analyses of subgroups and interactions, and sensitivity analyses	16
**Discussion**			
Key results	18	Summarise key results with reference to study objectives	16-18
**Limitations**			
Interpretation	20	Give a cautious overall interpretation of results considering objectives, limitations, multiplicity of analyses, results from similar studies, and other relevant evidence	17-19
Generalisability	21	Discuss the generalisability (external validity) of the study results	17-19
**Other information**			
Funding	22	Give the source of funding and the role of the funders for the present study and, if applicable, for the original study on which the present article is based	20

*Give information separately for cases and controls in case-control studies and, if applicable, for exposed and unexposed groups in cohort and cross-sectional studies.

Note: An Explanation and Elaboration article discusses each checklist item and gives methodological background and published examples of transparent reporting. The STROBE checklist is best used in conjunction with this article (freely available on the Web sites of PLoS Medicine at http://www.plosmedicine.org/, Annals of Internal Medicine at http://www.annals.org/, and Epidemiology at http://www.epidem.com/). Information on the STROBE Initiative is available at www.strobe-statement.org.

For peer review only
